# A robust multiplex immunofluorescence and digital pathology workflow for the characterisation of the tumour immune microenvironment

**DOI:** 10.1002/1878-0261.12764

**Published:** 2020-09-01

**Authors:** Amélie Viratham Pulsawatdi, Stephanie G. Craig, Victoria Bingham, Kris McCombe, Matthew P. Humphries, Seedevi Senevirathne, Susan D. Richman, Phil Quirke, Leticia Campo, Enric Domingo, Timothy S. Maughan, Jacqueline A. James, Manuel Salto‐Tellez

**Affiliations:** ^1^ Patrick G Johnston Centre for Cancer Research Queen's University Belfast Belfast UK; ^2^ Leeds Institute of Medical Research at St James's University of Leeds Leeds UK; ^3^ CRUK/MRC Oxford Institute for Radiation Oncology Oxford University Oxford UK; ^4^ Belfast Health and Social Care Trust Belfast UK

**Keywords:** image analysis, multiplex immunofluorescence, opal methodology

## Abstract

Multiplex immunofluorescence is a powerful tool for the simultaneous detection of tissue‐based biomarkers, revolutionising traditional immunohistochemistry. The Opal methodology allows up to eight biomarkers to be measured concomitantly without cross‐reactivity, permitting identification of different cell populations within the tumour microenvironment. In this study, we aimed to validate a multiplex immunofluorescence workflow in two complementary multiplex panels and evaluate the tumour immune microenvironment in colorectal cancer (CRC) formalin‐fixed paraffin‐embedded tissue. We stained CRC and tonsil samples using Opal multiplex immunofluorescence on a Leica BOND RX immunostainer. We then acquired images on an Akoya Vectra Polaris and performed multispectral unmixing using inform. Antibody panels were validated on tissue microarray sections containing cores from six normal tissue types, using qupath for image analysis. Comparisons between chromogenic immunohistochemistry and multiplex immunofluorescence on consecutive sections from the same tissue microarray showed significant correlation (*r*_s_ > 0.9, *P*‐value < 0.0001), validating both panels. We identified many factors that influenced the quality of the acquired fluorescent images, including biomarker co‐expression, staining order, Opal‐antibody pairing, sample thickness, multispectral unmixing and biomarker detection order during image analysis. Overall, we report the optimisation and validation of a multiplex immunofluorescence process, from staining to image analysis, ensuring assay robustness. Our multiplex immunofluorescence protocols permit the accurate detection of multiple immune markers in various tissue types, using a workflow that enables rapid processing of samples, above and beyond previous workflows.

## Graphical abstract

Optimisation and validation of a multiplex immunofluorescence (mIF) workflow, from staining to digital image analysis (DIA), ensure assay robustness. Chromogenic immunohistochemistry (IHC) and fluorescent singleplexes are fundamental in this process, particularly when biomarkers are co‐expressed. We describe our experience developing two mIF panels and the various parameters of staining, scanning and DIA to consider when standardising a digital pathology workflow.

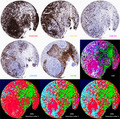

**Abbreviations**
CK: cytokeratin
CRC: colorectal cancer
DAB: 3,3′‐diaminobenzidine
DIA: digital image analysis
ER: epitope retrieval
FFPE: formalin‐fixed paraffin‐embedded
HIER: heat‐induced epitope retrieval
IF: immunofluorescence
IHC: immunohistochemistry
mIF: multiplex immunofluorescence
MP: multiplex panel
ROI: regions of interest
S:CORT: Stratification for Colorectal Cancer Consortium
TMA: tissue microarray
TSA: tyramide signal amplification

## 1 Introduction

Opal multiplex immunofluorescence (mIF) is based on the principle of tyramide signal amplification (TSA), wherein fluorescent Opal dyes are conjugated with tyramide molecules to produce enzymatic amplification, similar to conventional chromogenic immunohistochemistry (IHC) [1, 2]. This enables investigators to more accurately detect the presence of infrequently expressed biomarkers and permits the use of well‐validated primary antibodies raised in the same species [3]. Application of these assays was previously limited to small‐scale studies since quantitative analysis of whole‐slide mIF staining in large cohorts was rendered unfeasible by the lack of assay automation, whole‐slide imaging and dedicated image analysis software [4]. Today, however, there exist autostainers capable of automating mIF protocols with quick turnaround times. Such protocols would have taken up to a week to conduct manually, increasing the likelihood of batch effects and human error [5]. In addition, slide scanners now offer the possibility of fluorescent whole‐slide image capture. The resultant multilayered pyramidal tiff files can be easily processed using commercial and open‐source software that are now available for comprehensive digital image analysis (DIA) [1, 6].

The ability to use standardised DIA algorithms for the assessment of chromogenic IHC staining has been markedly improved by the use of validated, automated staining protocols, standardised whole‐slide scanning and image processing [1, 7, 8]. In essence, optimisation of the entire pathology workflow is key in determining assay robustness and troubleshooting efficiency [2]. The possibility of validating automated mIF assays therefore brings the prospect of deploying the technique into clinical practice [5]. However, it is not yet known how considerations during multiplex optimisation, such as Opal‐antibody pairings and multispectral unmixing, affect the standardisation of image analysis and digital pathology.

In this study, we describe our experience validating a digital pathology workflow for which two complementary mIF assays were developed. We evaluate how mIF compares to traditional IHC for biomarker quantification, following assay optimisation based on relative epitope stability, optimal antibody concentrations, antibody staining order, Opal‐antibody pairings and denaturing protocols. Furthermore, we assess whether factors affecting mIF image acquisition, such as co‐expressing biomarkers, crosstalk and relative fluorophore intensity, need to be considered when standardising DIA algorithms. The two multiplex panels were therefore designed to include co‐expressing biomarkers to assess the impact of cellular co‐expression on DIA. Multiplex panel one (MP1) was designed as a 6‐plex assay, comprising CD3, CD4, CD8, CD20, cytokeratin (CK) and DAPI, with CD3 expected to colocalise with CD4 and CD8 in the membrane of T cells. Multiplex panel 2 (MP2) was created as a 5‐plex assay, consisting of CD4, CD68, FOXP3, CK and DAPI. This time assesses the effect of two biomarkers, CD4 and FOXP3, colocalising in different cellular compartments of regulatory T cells. In addition, we investigate the importance of pre‐analytical factors in DIA such as sample thickness, multispectral unmixing and batch versus individual scan exposures to assess how they may affect the acquired images and subsequent analysis. Using these data, we conclusively determine how important the technical challenges unique to mIF can influence the robustness of a DIA protocol.

## 2 Materials and methods

### 2.1 Patient samples

The study was conducted according to the Good Clinical Practice guidelines and the Declaration of Helsinki. All patients provided informed consent for sampling of their tissue. Protocol optimisation was carried out on full‐face tonsil and colorectal resection specimens provided as 4 µm, 10% neutrally buffered, formalin‐fixed paraffin‐embedded (FFPE) tissue sections. Access to these slides was granted under the NIB approval (OREC 16/NI/0030; NIB15/0168). Ethical approval for use of these samples in research was granted through the Northern Ireland Biobank (NIB15/0168). The optimised mIF protocols were validated against chromogenic singleplex protocols on consecutive sections of a tissue microarray (TMA). The TMA was constructed with 4 × 0.6 mm tissue cores taken from normal tonsil, placenta, colonic epithelium, lymph node, appendix and spleen using a Beecher manual arrayer (Beecher Instruments Inc., Sun Prairie, WI, USA). Both validated mIF protocols were then tested on full‐face resection specimens from the tissue of interest, colorectal cancer (CRC). Access to these slides was granted under collaboration with the Stratification for Colorectal Cancer Consortium (S:CORT) with appropriate approvals in place (OREC 15/EE/0241). Ethical approval for use of these samples in research was granted through NHS REC proportionate review (OREC 15/EE/0241). All work on the tissue sections was performed in the Precision Medicine Centre of Excellence at Queen's University Belfast using standardised operating procedures for IHC staining, digital slide scanning and DIA. All procedures were reviewed and agreed by senior consultant pathologists (JAJ and MS‐T).

### 2.2 Tissue preparation for staining

Prior to staining, all tissue slides were deparaffinised on the Leica BOND RX automated immunostainer (Leica Microsystems, Milton Keynes, UK) by baking for 30 min at 60 °C, soaking in BOND Dewax Solution at 72 °C and then rehydrating in ethanol.

### 2.3 Chromogenic singleplex immunohistochemistry

Singleplex IHC using 3,3′‐diaminobenzidine (DAB) detection (BOND Polymer Refine Detection, Leica Biosystems, Newcastle Upon Tyne, UK; Catalogue No. DS9800) was carried out to determine the conditions and the order in which the primary antibodies would be applied in the multiplex protocol. To ensure adequate epitope stability following successive rounds of heat‐induced epitope retrieval (HIER), chromogenic singleplex IHC was conducted in the first, intermediate and last round of HIER for each of the biomarkers to be multiplexed in MP1, corresponding to positions 1, 3 and 5 of antibody staining (Data S1). Results of positions 3 and 5 reflected those of positions 2 and 4, respectively, which permitted rapid IHC optimisation. Staining was performed on the Leica BOND RX with HIER pretreatments applied at 95 °C using BOND Epitope Retrieval (ER) Solutions: citrate‐based pH 6.0 ER1 (Leica Biosystems; Catalogue No. AR9961) or EDTA‐based pH 9.0 ER2 (Leica Biosystems; Catalogue No. AR9640).

### 2.4 Singleplex and multiplex immunofluorescence

The TSA‐based Opal method was used in this study for immunofluorescence (IF) staining (Opal Polaris 7‐Color Automation IHC Kit; Akoya Biosciences, Marlborough, MA, USA; Catalogue No. NEL871001KT). Since TSA and DAB oxidation are both peroxidase‐mediated reactions, the primary antibody conditions and order of staining determined using DAB detection were directly applied to the fluorescent assays, using Opal reagents in lieu of DAB IHC reagents (Data S2). Unlike conventional IHC wherein a chromogenic peroxidase substrate is used for antigen detection, each antibody is paired with an individual Opal fluorophore for visualisation. Optimal Opal‐antibody pairings were assigned based on expected co‐expression and abundance of the biomarkers in CRC tissue. More explicitly, if biomarkers were expected to colocalise in the same cellular compartment, then they were paired with spectrally separated Opals. Additionally, low‐expressing markers were coupled to more intense Opals to facilitate spectral acquisition, and vice versa. The Opal fluorophores were used at a 1 in 150 dilution, as recommended by Akoya when using the Leica BOND RX. As such, a fluorescent singleplex was performed for each biomarker and compared to the appropriate chromogenic singleplex to assess staining performance. To confirm the absence of signal crosstalk, a dropout control was ran for each antibody using the final optimised multiplex [9].

During singleplex development and multiplex optimisation, Opal‐antibody pairings, concentrations and denaturing parameters for each biomarker were assessed and adjusted for. This was done by checking the signal‐to‐background ratio (signal intensity of positive staining: background > 10 : 1) and signal balance (signal intensity of all fluorophores < 30 counts) with Akoya's inform software version 2.4.6. We aimed at obtaining the ideal signal intensity range of 20–25 counts for each antibody, which translates as 100–125 nm of fluorescence capture on the Vectra Polaris [7]. All Opal dyes were initially considered for Opal‐antibody pairings, but along the optimisation process we transitioned to using only MOTiF Opals to take advantage of the MOTiF technology that enables rapid 7‐colour whole‐slide multispectral imaging of fluorescent slides.

### 2.5 Image acquisition

All chromogenic immunostained slides were scanned using an Aperio AT2 (Leica Biosystems, Vista, CA, USA) at 40× magnification and then were independently reviewed for quality and consistency by a trained senior technician (VB) before being considered for DIA.

All fluorescently labelled slides were scanned on the Vectra Polaris at 20× magnification using appropriate exposure times. Initially, whole‐slide images were scanned with all five standard epi‐fluorescence filters (DAPI, FITC, Cy3, Texas Red and Cy5). Then, when MOTiF Opals were solely used, images were acquired using tile scanning with the 7‐colour whole‐slide unmixing filters (DAPI + Opal 570/690, Opal 480/620/780 and Opal 520). Library slides were generated from representative tissue sections to allow for accurate unmixing of the multiplexed samples, including a slide stained for each single fluorophore, a DAPI only slide and an autofluorescence slide wherein no antibody, Opal reagent or DAPI was applied. The unmixing performance of this tissue‐specific spectral library was compared to that of the synthetic Opal library available in inform. Resultant image tiles were then stitched together within qupath v0.2.0‐m4 using a script available on the qupath GitHub to produce a whole‐slide multichannel, pyramidal OME‐TIFF image for DIA [10].

### 2.6 Digital image analysis

Assessment of all biomarkers was undertaken using the open‐source software qupath v0.2.0‐m4. Each core of the TMA, used for validation, was given a unique identifier in order to directly compare cellular expression between slides. The full‐face sections, used for optimisation and testing, were annotated with the assistance of a senior consultant pathologist (MS‐T). Following tissue annotation, cell segmentation was carried out based on haematoxylin if chromogenic or DAPI if fluorescent. Phenotyping of the cells was then performed using binary classification in the chromogenic images, or using a bespoke script enabling multimarker cataloguing of detected cells in the fluorescent images. All analysed images were independently reviewed for quality control purposes, with at least 20% being reviewed by a pathologist prior to data export.

During the progress of this study, the latest qupath milestone v0.2.0‐m9 was released, offering a new approach for multiplex analysis. We analysed the multiplexed TMA sections, used for validation, in this latest version of qupath, which provides two methods of mIF classification. Method 1 involves simple thresholding of a single measurement to classify each biomarker, similar to the approach used in v0.2.0‐m4. Method 2 utilises a machine learning classifier, random forest, which is trained on multiple measurements. Once the classifiers have been established for each biomarker using either method 1 or 2, these are combined and applied sequentially. The newly generated phenotype results were compared to those previously obtained with qupath v0.2.0‐m4.

### 2.7 Statistical analysis

All statistical analyses were conducted using prism version 5 (Graphpad Software, San Diego, CA, USA). The relationship between the staining parameters (antibody order, Opal‐antibody pairing) and the percentage of positive cells detected was assessed using the Mann–Whitney *U*‐test. For validation purposes, a difference in cell positivity of greater than 10% (i.e., less than 90% accuracy of obtaining a similar result) between DAB and IF cell counts was considered significant. The monotonic relationship between the singleplex IHC and mIF was assessed using Spearman's rank correlation. *P*‐values of less than 0.05 were considered significant. The agreement between the singleplex IHC and mIF assays was assessed using a Bland–Altman plot, and the determinant bias was considered acceptable if within the 95% limits of agreement. Comparison of the qupath v0.2.0‐m4 and ‐m9 data was conducted using a paired *t*‐test, with a two‐tailed *P*‐value of less than 0.05 defined as being significant.

## 3 Results

### 3.1 Chromogenic singleplex optimisation

**Fig. 1:**
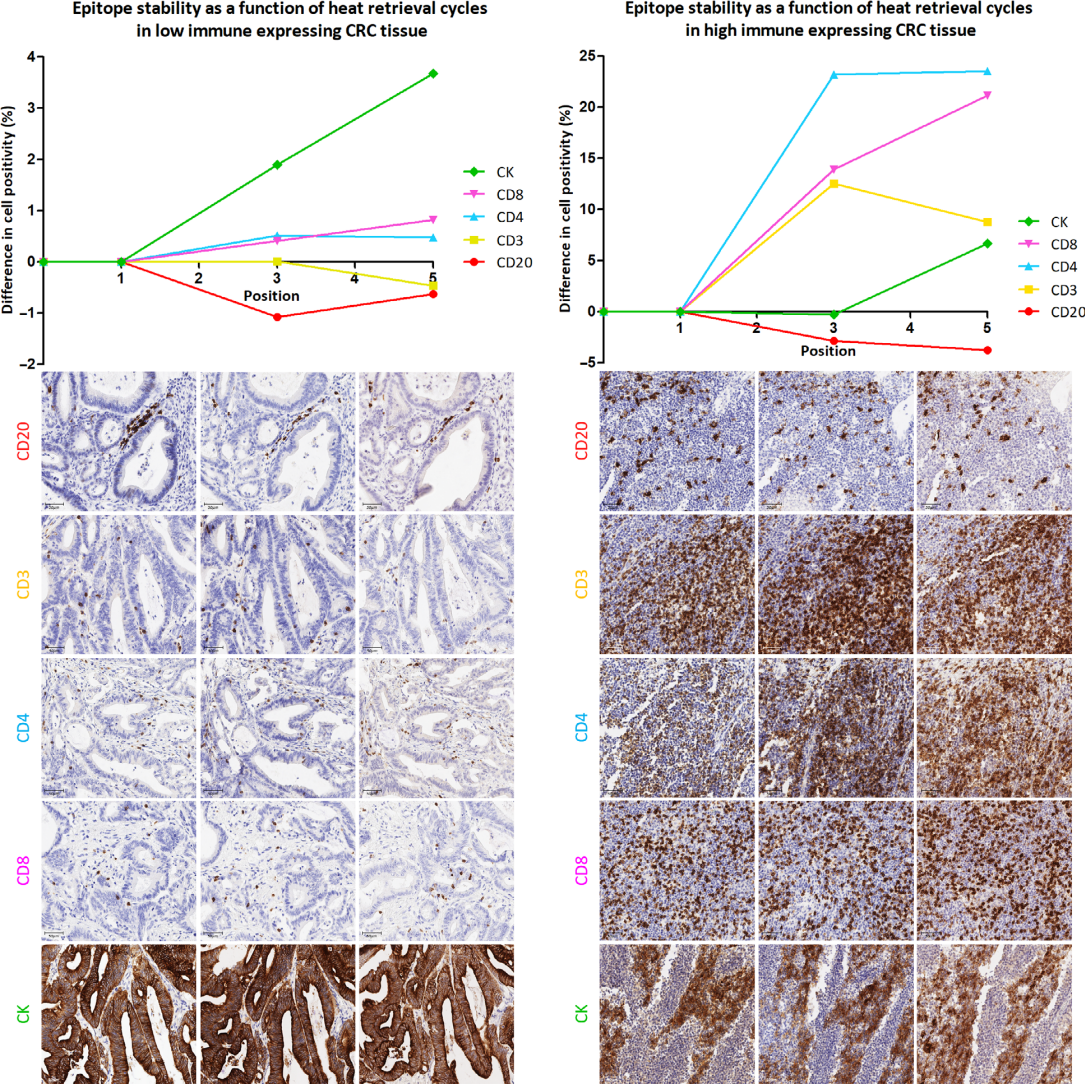
Epitope stability as a function of antibody positioning in MP1. Line graphs depicting how biomarker detection in low (left) and high (right) immune expressing CRC tissue changes according to the number of HIER cycles preceding antibody application, that is the position of an antibody in a multiplex. Fifteen chromogenic singleplexes were performed on each of the two CRC cases (*n* = 30). The corresponding images are shown below each graph: position 1 (left), position 3 (middle) and position 5 (right). Images were scanned at 40× magnification and are displayed at 20× magnification (scale bar = 50 µm).

Singleplex development is fundamental in designing a multiplex assay as it determines the staining parameters for each individual biomarker in the multiplex panel. In order to identify the best placement of each biomarker in the staining sequence for MP1, three chromogenic IHC singleplexes for each primary antibody were performed on serial sections of low and high immune expressing CRC tissue. Epitope stability was evaluated as a function of the number of HIER cycles preceding antibody application (Fig. 1). Stability was defined by the absence of relative change in cell positivity across the serial sections. Epitope denaturation, on the other hand, was represented by a decline in staining intensity and consequently a drop in positive cells detected. In the current study, CD20 was found to be an epitope requiring minimal retrieval and was therefore best placed in position 1. Results also showed CD3 required limited retrieval and could be placed in position 2, whereas CD4, CD8 and CK exhibited an increase in cell positivity with continued epitope retrieval, and were therefore best placed in positions 3–5.

### 3.2 Fluorescent singleplex optimisation

**Fig. 2:**
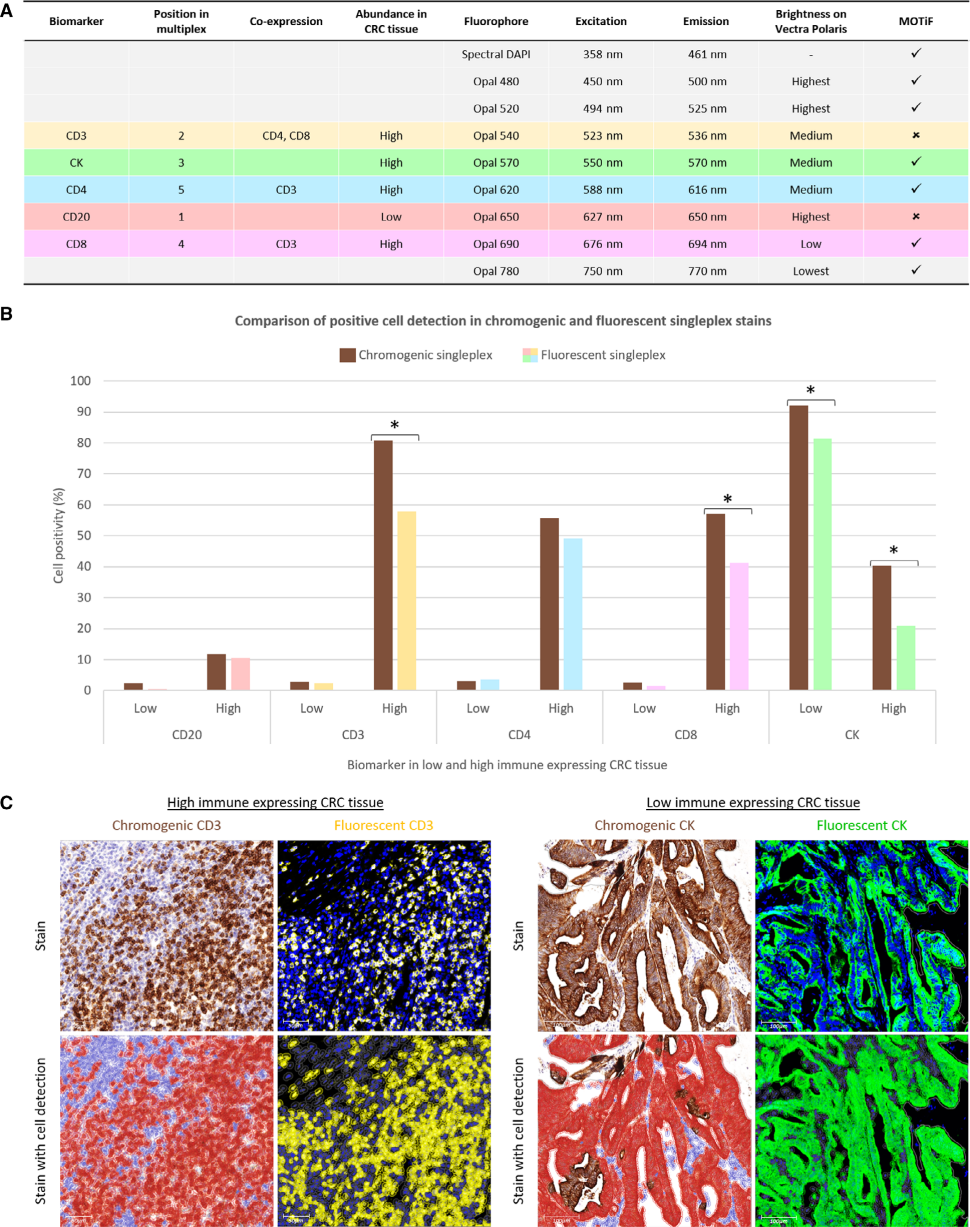
Fluorescent singleplex development and comparison to chromogenic singleplex. (A) MP1 Opal‐antibody pairings based on the position, colocalisation and expected abundance of the biomarkers (columns 1–5). Columns 6–8 present the spectral properties of the fluorophores and the last column the fluorophores that qualify for MOTiF scanning. (B) Fluorescent singleplexes (*n* = 10) were compared to the chromogenic singleplexes (*n* = 10) previously performed on the same low and high immune expressing CRC tissue. Bar graph demonstrating the percentage of cell positivity for each biomarker in low and high immune expressing CRC tissue when stained with DAB (brown) versus Opal (multicolour) reagents. Statistical significance was determined by the Mann–Whitney *U*‐test. Difference of > 10% in cell positivity in four cases, marked with an asterisk. (C) ROI of two of these cases is displayed. For each case, the chromogenic stain is seen on the left and fluorescent stain on the right, with the original image above and with cell detection applied below. The CD3 stains are viewed at 20× magnification (scale bar = 50 µm), and the CK stains are seen at 10× magnification (scale bar = 100 µm). In the chromogenic CD3 stain, there are more positive cells (in brown) than there are in the fluorescent CD3 stain (in yellow), which is reflective of the different immune expression profiles that exist between nonserial sections. In the chromogenic CK stain, some DAB‐positive cells are not detected by qupath as being a negative cell (blue) nor a positive cell (red). However, all cells are identified in the fluorescent CK stain as being either negative (grey) or positive (green).

Based on the visual assessment and quantitative analysis of the chromogenic singleplex stains, the preliminary order of MP1 staining consisted of CD20 in position 1, followed by CD3, CK, CD8 and CD4 (Fig. 2A). Taking into consideration biomarker co‐expression and relative abundance, initial Opal‐antibody pairings were assigned to each biomarker based on the chromogenic singleplex results. CD4 and CD8 were coupled to fluorophores spectrally distant from the CD3‐associated Opal, in addition to them being sequentially separated from CD3 (permitted by assigning CK to position 3). CD3, CD4, CD8 and CK were all highly expressing targets in the representative tissue type, so they were coupled to low‐ or medium‐intensity Opals. CD20, which was found to be low expressing, was paired with a brighter fluorophore (Opal 650).

The fluorescent singleplexes were performed on the same low and high immune expressing CRC cases as the chromogenic singleplexes to permit comparison. Positive cell detection in similar regions of interest (ROI) demonstrated less than 10% difference in all but four cases: CD3, CD8 and CK in high immune CRC; CK in low immune CRC (Fig. 2B). These significant differences are reflective of the variant cellular profiles that exist among nonserial sections. Review of these cases found that such differences were also attributed to qupath's inability to accurately quantify cells in regions with dense populations of DAB‐positive cells (Fig. 2C). DAB is an opaque stain; therefore, colour deconvolution in these intensely stained samples was inept in separating the haematoxylin channel for accurate cell detection [2].

### 3.3 Multiplex immunofluorescence optimisation

With optimal staining parameters established for each biomarker, creation of a spectral unmixing library, imperative to the accurate analysis of the forthcoming multiplex slides, was required. While the manufacturer's guide recommends building library slides specific to the tissue type of the study, we anticipated many amendments to the Opal‐antibody pairings during multiplex optimisation, which would require a new library to be built each time [9]. As a means to replace this step, we compared the spectra and unmixing performances of our newly created CRC‐specific library and the synthetic Opal library (constructed from tonsil tissue) (Data S3). No significant differences were observed, thereby validating the use of the synthetic library moving forward.

**Fig. 3:**
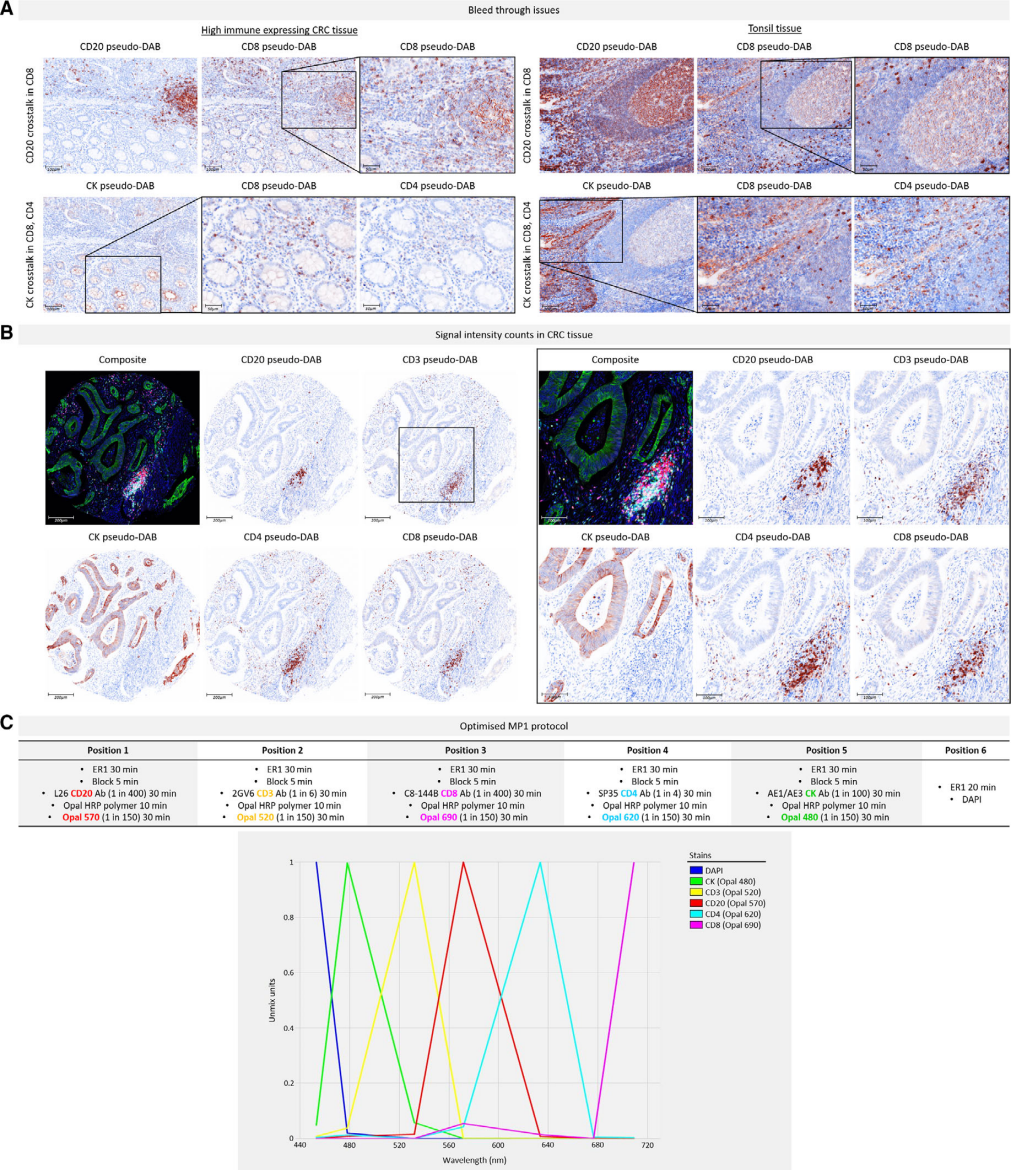
Overview of MP1 optimisation. (A) Example images viewed in inform as simulated DAB IHC images (‘pseudo‐DAB’) to show the bleed through issues encountered during MP1 protocol optimisation, in CRC tissue (left) and tonsil tissue (right). Images are displayed at 10× magnification (scale bar = 100 µm) and magnified fields of view (in the black boxes) at 20× magnification (scale bar = 50 µm). Top row: CD20 crosstalk in the CD8 channel. Bottom row: CK crosstalk in the CD8 and CD4 channels. The CK crossover into CD8 is more evident since CD8 was positioned directly after CK in this early MP1 protocol (staining order CD20 > CD3 > CK > CD8 > CD4). (B) Example images of a CRC core stained with a more developed MP1 protocol, following resolution of the bleed through issues. Magnified images of the same core (within the black box) highlight the clean staining obtained for each marker. Images are viewed at 10× magnification (scale bar = 200 µm) and magnified fields of view at 20× magnification (scale bar = 100 µm). All signal intensity counts are within the acceptable range of 20–25 except for CD20 (43.6), but this was resolved in the final protocol. (C) The optimised MP1 protocol with the spectrum of the MOTiF Opals used below. No overlap is seen between the emission peaks. Obtaining the final optimised MP1 protocol required the development of nine protocols and the use of *n* = 40 tissue sections.

The fluorescent singleplex protocols were combined into an initial multiplex protocol, which was progressively modified following the manifestation of spectral bleed through (Fig. 3A). Most predominantly, CD20 crosstalk was observed in the CD8 channel, in both CRC and tonsil tissue. This was initially explained by the use of CD20‐Opal 650 and CD8‐Opal 690 pairings as both Opals are acquired in the Cy5 channel, within which their emission peaks overlap (Data S3). By changing the Opal‐antibody pairings, the possibility of spectral crosstalk was eliminated, yet crossover remained present in immune hot spots, that is germinal centres. Dropout controls confirmed that the observed crosstalk was not due to inadequate antibody stripping (Data S4). As CD8 was placed in position 3 of MP1, we determined that epitope instability may be inducing this effect [8]. However, due to the inclusion of less stable HIER antibodies CD20 and CD3 in MP1, we could not modify the staining position of CD8. The other issue originally observed was CK bleed through into CD8 and CD4 channels, which was easily addressed by reordering the biomarkers.

In addition to revising Opal‐antibody pairings, positions and dilutions, there were two major changes made during mIF protocol optimisation. The first was a switch to using solely ER1 for antibody stripping. Because ER1 is a gentler epitope retrieval method, using a pH of 6 as opposed to a pH of 9 in ER2, it generates less nonspecific staining and background signal. The second amendment was a switch to using only MOTiF Opals for signal amplification. MOTiF Opals are a preferred choice as they allow scanning times 20× quicker than was formerly possible, owing to the fact that they are excited by filters located on merely three filter wheels in the Vectra Polaris (only available with the upgraded filter cube for 7‐colour whole‐slide unmixing) [11]. Moreover, MOTiF Opals have spectrally distant peaks that permit efficient multispectral unmixing.

Subsequent to each adjustment made in the protocol, signal‐to‐background ratio and signal balance for each revisited Opal‐antibody pairing were reassessed. Optimal signal intensities of 20–25 counts were achieved as the protocol developed. Figure 3B shows example images of a CRC TMA core stained with one of these later protocols, and Fig. 3C presents the final optimised MP1 protocol. The diligent optimisation process of MP1, requiring the trial of numerous protocols, enabled the rapid development of MP2 (Data S5). Essentially, experience with antibody behaviour and Opal methodology facilitated the creation of new panel designs quickly.

### 3.4 Optimisation of high‐throughput image acquisition

**Fig. 4:**
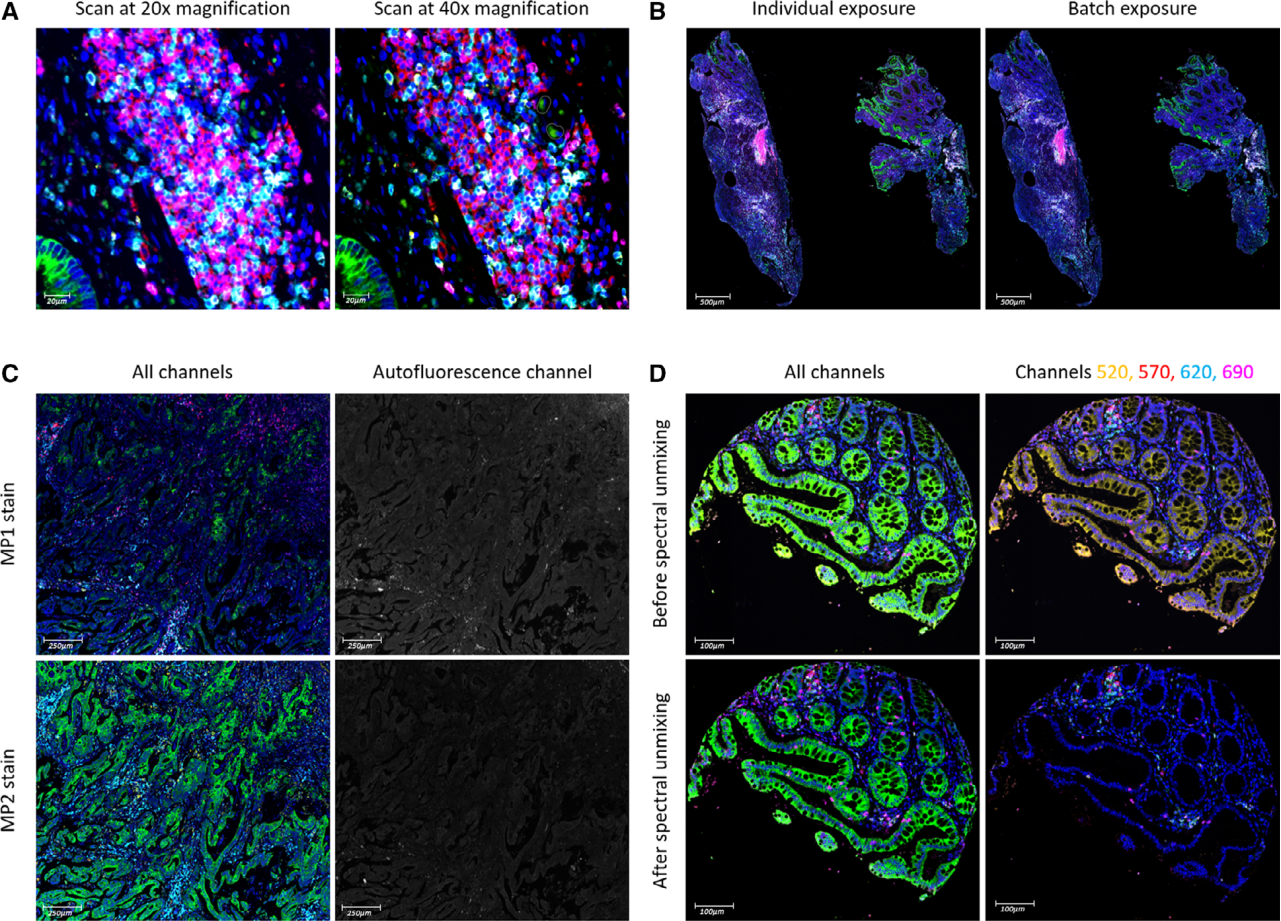
Factors considered for DIA. (A) A full‐face CRC section was scanned at 20× and 40× magnification. The same region, viewed at 40× magnification (scale bar = 20 µm), was selected on each scan. The only notable difference is the higher resolution of the 40× scan. (B) Full‐face CRC sections stained with MP1 (*n* = 5) were scanned using individual and batch exposure times. The scans of one of these samples are shown here at 1.5× magnification (scale bar = 500 µm). Spearman's rank correlation determined that no difference exists in their staining intensities. (C) Two serial full‐face CRC sections were stained, one for MP1 (top row) and one for MP2 (bottom row). The difference in their staining intensities is seen in the left column: CK (green) and CD4 (cyan), which are present in both panels, are more intense in MP2 than in MP1. This originates from the difference in their paraffin section thickness, seen in the right column via the autofluorescence channel: Autofluorescence in MP1 is greater than in MP2 on account of the MP1 section being thicker than the MP2 section. Images are viewed at 5× magnification (scale bar = 250 µm). (D) A colonic core before (top row) and after (bottom row) spectral unmixing in inform. The epithelium is expected to stain green for CK (Opal 480) and not yellow for CD3 (Opal 520). Other MP1 markers seen here are CD20 in red (Opal 570), CD4 in cyan (Opal 620) and CD8 in magenta (Opal 690). Images are viewed at 10× magnification (scale bar = 100 µm).

The Vectra Polaris allows high‐speed digital whole‐slide scanning at 10×, 20× and 40× magnification in both brightfield and fluorescence. Once the final multiplex protocols (MP1 and MP2) were realised, 20× and 40× fluorescent scanning were tested on a MP1‐stained CRC full‐face sample (Fig. 4A). The high resolution in the 40× scan offered a more defined image of the cells and their surface boundaries than the 20× scan. However, there was less than 1% difference in the cell populations detected and such detail was not considered essential for the purpose of this validation study (Data S6). So with the advantage of minimised scanning time, 20× scanning was selected hereafter.

When setting exposure times for image acquisition, the manufacturer's guide recommends autoexposing on several slides in a batch for each fluorophore [9]. This method of batch exposure was compared to individual exposure, wherein the channels were autoexposed for each individual slide, thus generating slide‐specific scanning protocols. Once scanned, a single autofluorescence image was used for spectral unmixing. Results revealed no difference in staining intensities, both visually and quantitatively (*r*_s_ > 0.9, *P* < 0.05) (Fig. 4B). So with it requiring less preparation time, batch exposure was applied moving forward.

Sample thickness, on the other hand, was found to be a determining factor in producing reliable staining intensities. Although tissue blocks were cut with the intention of generating regular 4‐µm sections, we encountered intersection thickness variability during mIF optimisation, which occurs as a result of thermal expansion in microtomy [12]. For instance, two serial full‐face CRC sections were stained with MP1 and MP2 (Fig. 4C). Both comprising of CD4 and CK, the panels were expected to produce similar expression patterns for helper T cells and epithelial tumour cells. However, staining was considerably weaker in the MP1 scan compared to the MP2 scan, which was due to the MP1‐stained tissue being thicker than the MP2‐stained tissue. Since FFPE tissue is inherent to autofluorescence, and autofluorescence signal intensity is positively correlated with tissue thickness, viewing the images in the autofluorescence channel enabled visual comparison of their section thickness [13, 14]. The MP1‐stained section was notably brighter in autofluorescence intensity and hence thicker. Evidently, regular thin cuts of tissue with a thickness of 3–4 µm are considered most suitable for immunofluorescence [7, 15].

Following image acquisition, it was essential to import the scanned images into inform for spectral unmixing, which included removing autofluorescence using a representative autofluorescence spectrum [2, 9]. We tested the importance of unmixing by importing scans directly into qupath from the scanner. The channels remained mixed and the autofluorescence present. As a result, there were high levels of nonspecific staining, which rendered the determination of certain biomarker thresholds and subsequent cell classification impossible. As seen in Fig. 4D, the most apparent nonspecific staining was associated with Opal 520 (assigned to CD3), which emits in the FITC channel [16]. Consequently, the colonic epithelium appears to be staining for CD3. As an alternative workflow, the same scans were first opened in the whole‐slide viewer phenochart v1.0.12 (Akoya Biosciences, Marlborough, MA, USA) to select for ROI, imported into inform for unmixing using the synthetic library and then stitched together in qupath. The same example image, now unmixed, no longer appears to be staining for CD3 in the epithelium. The substantial difference in image quality therefore confirms that this workflow is necessary for DIA in qupath.

### 3.5 Validation of high‐throughput multiplex immunofluorescence protocols

**Fig. 5:**
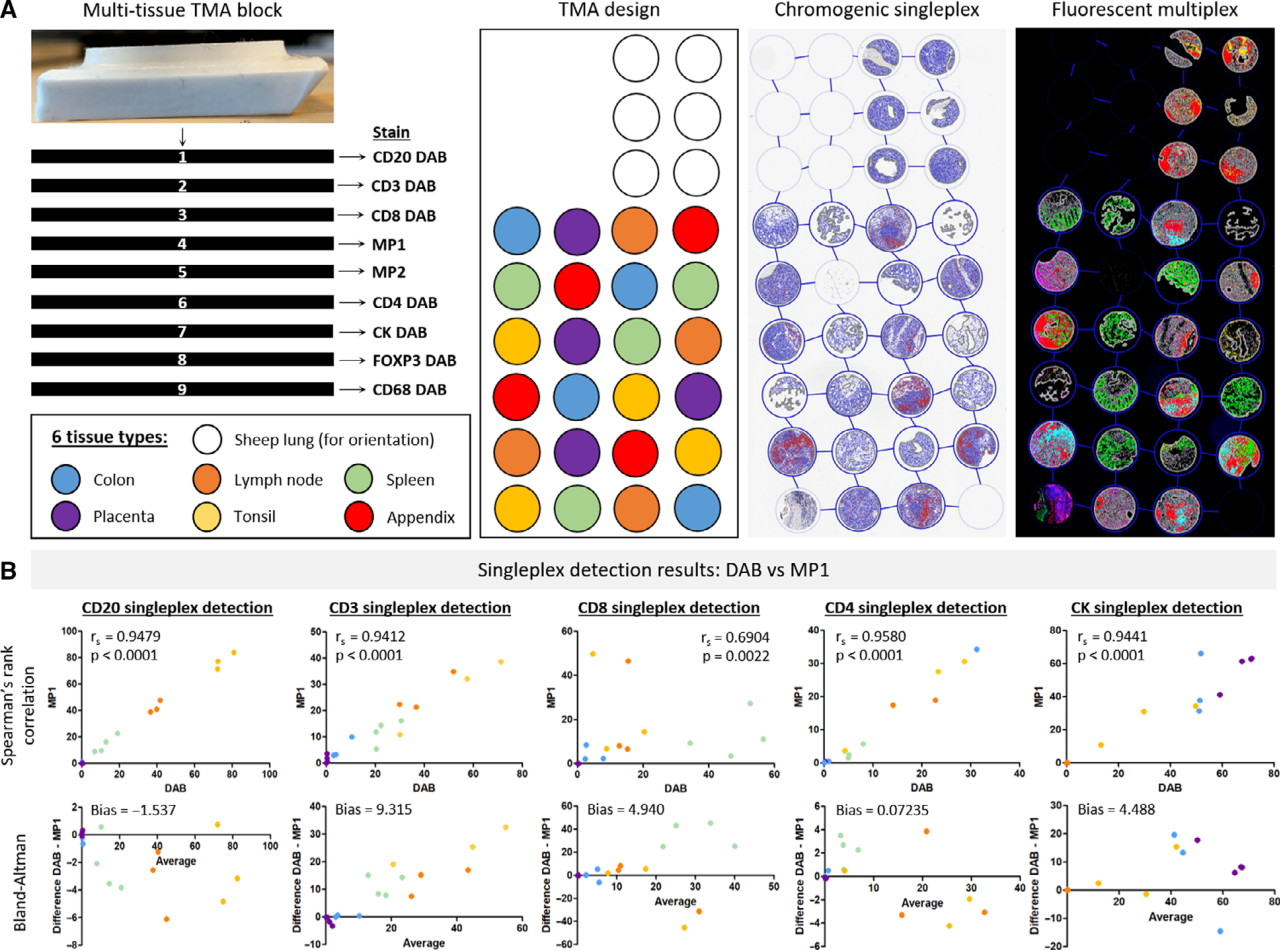
Overview of mIF validation. (A) Nine consecutive sections were cut from a TMA block, of which seven were stained for single antibody DAB IHC and two for mIF. Section 4 stained for MP1 and section 5 stained for MP2. Three right images depict overviews of the TMA layout, a chromogenic TMA and a multiplexed TMA, with tissue annotation and cell detection applied in the latter two. The TMA consists of six normal human tissue types, plus sheep lung tissue for orientation. (B) MP1 validation results. Spearman's rank correlation graphs (top) and Bland–Altman plots (bottom) illustrating the relationship and agreement between DAB detection (from TMAs 1, 2, 3, 6, 7) and singleplex IF detection (from TMA 4), for each biomarker in MP1. The same cores (*n* = 17) across all the TMAs were used for analysis of the biomarkers. All correlations are strong (*r*_s_ > 0.9, *P* < 0.0001) except for CD8, and all biases are within the limits of agreement.

Having fully optimised two panels and implemented a streamline workflow from staining to acquiring quality digital images, the next stage was to validate the protocols and establish a process for analysing the fluorescent images. In the first instance, a TMA block comprising of six normal tissue types was cut into nine consecutive sections. Of these, seven sections were chromogenically stained with a single antibody, one slide for each different biomarker, leaving two sections to be stained with the optimised multiplex panels (Fig. 5A). The chromogenic TMAs were annotated in qupath, and cell detection was carried out using appropriate DAB thresholds. The multiplexed TMAs were annotated in the same way, mimicking the chromogenic ROI as much as possible. Cell classification was done by meticulously determining thresholds for each marker, analogous to the DAB thresholds. Each phenotype was initially identified as a ‘singleplex detection’, wherein one marker was exclusively detected across the entire multiplexed TMA, for example CD20‐positive cell detection on MP1 TMA. These ‘singleplex’ results were compared to the DAB results of the consecutive sections to assess biomarker correlation and biasness (Fig. 5B for MP1; Data S7 for MP2).

Cell detection results of all the chromogenic TMAs (sections 1, 2, 6 and 7) correlated significantly with the singleplex detection data from MP1 TMA (section 4) (*r*_s_ > 0.9, *P* < 0.0001), except for CD8 (TMA section 3). With the latter being nearest to the MP1 TMA section in terms of depth level in the original TMA block, one would expect their immune expression profiles and therefore their detection results to be most comparable. Nonetheless, the poor correlation and high biasness appeared to be driven by three tissue types: spleen, tonsil and lymph node. In spleen, the histiocytic littoral cells that are highly positive for CD8 were detected by DAB staining but not by mIF. In tonsil and lymph node tissue, the CD8 positivity associated with CD20^+^ germinal centres was picked up by mIF but not by conventional IHC, supporting our conclusion of reduced epitope stability (Data S8). Similarly, cell detection results for MP2 (TMA section 5) displayed strong correlation for all but one biomarker, FOXP3 (TMA section 8) (*P* > 0.05), which is likely due to the protein being scarcely expressed in the TMA (< 0.5% expression). Meanwhile, any biasness observed among the other biomarkers was explained by the occurrence of intense DAB staining affecting accurate cell detection. Overall, all determinant biases fell within the limits of agreement and there was significant positive correlation between the DAB and mIF stains, thus validating the multiplex protocols.

### 3.6 Optimisation of digital image analysis protocols

**Fig. 6:**
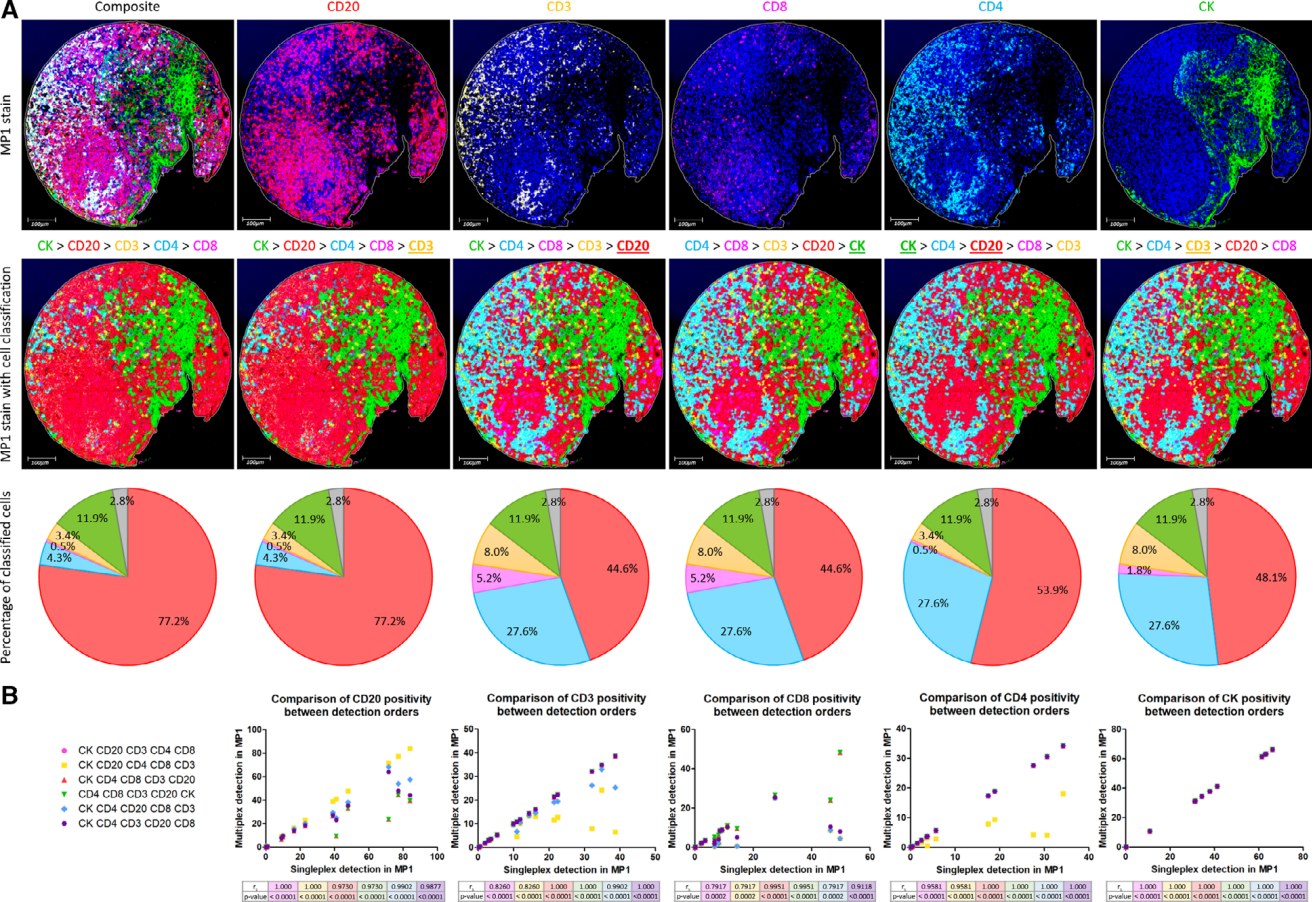
Importance of the detection order in digital assessment of MP1. (A) Example images of tonsil core 3 stained with MP1 protocol, seen at 10× magnification (scale bar = 100 µm). Top row shows the original stains: a composite image followed by an image for each individual marker. Middle row illustrates the same composite image to which six different detection orders were applied. From left to right, the accuracy of the detection orders increases. From one order to the next, the biomarker that has been modified is underlined. Bottom row presents the phenotypes identified by each detection order as a percentage of the classified cells. Pie chart colours are in accordance with cell classification colours above. Unclassified cells are indicated in grey. The final detection order (CK > CD4 > CD3 > CD20 > CD8) best represents the original stain visually. (B) Scatter graphs showing the correlation between each multiplex detection order and the singleplex detection of MP1 TMA 4, for each biomarker. The same cores (*n* = 17) of MP1 TMA 4 were used for analysis of the biomarkers. Statistical significance was measured by Spearman's rank correlation coefficient. The final detection order (purple) best matches the singleplex results quantitatively (*r*_s_ > 0.9, *P* < 0.0001 for all the biomarkers).

As well as running ‘singleplex detection’ on the multiplexed TMAs, cells were classified for all the phenotypes in a hierarchical manner. During this process, we found that the order of classification considerably influenced the phenotyping results for MP1, but not for MP2. Six detection orders were tested on MP1 TMA 4 (Fig. 6) and four were tried on MP2 TMA 5 (Data S9). As seen in Fig. 6, detecting strongly expressing targets early on in the classification order masks the subsequent detection of weaker, neighbouring biomarkers. This was particularly evident in immune hot spots such as germinal centres. By detecting membranous CD20 staining first, recognition of other membranous lymphocyte markers was hindered. As a result, CD20 was most suitable towards the end of the order but still placed before CD8 to avoid true B lymphocytes being classed as CD8^+^ cells, considering the nonspecific CD20‐CD8 staining present in these lymphoid follicles.

**Fig. 7:**
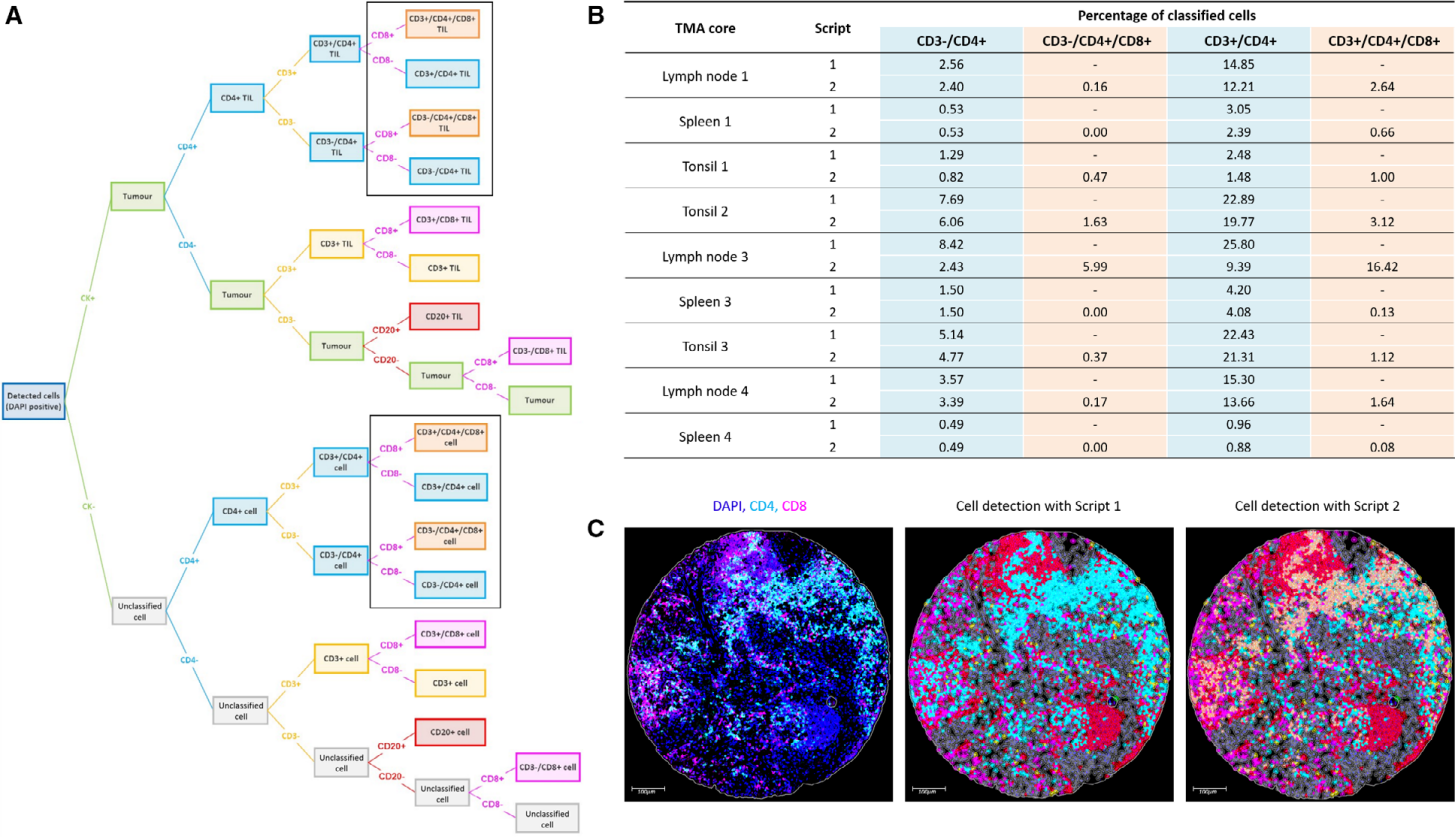
Cell classification in MP1 using a bespoke script. (A) Decision tree visually representing the coding instructions in the script. Unexpected classes are indicated within the black boxes. The original script did not contain these unexpected phenotypes, as opposed to the updated script. TIL, tumour‐infiltrating lymphocyte. (B) Differences in cell classification were only observed in the lymphoid tissue cores (lymph node, spleen and tonsil) (*n* = 9) when comparing the original script (script 1) and the updated script (script 2). Only a percentage of CD4^+^ cells that were found in hot immune regions are reclassified by script 2 as CD4^+^/CD8^+^ cells. (C) Example displaying the reclassification of these CD4^+^ cells (cyan) to CD4^+^/CD8^+^ cells (orange). Colours are in accordance with the colour‐coded phenotypes of the decision tree. Unclassified cells are indicated in grey. The core shown is of lymph node 3, seen here at 10× magnification (scale bar = 100 µm).

In order to facilitate the multimarker cataloguing of cells in these multiplexed TMAs, a script was created for each panel (Data S10 and S11). These scripts translate as decision trees (Fig. 7A; Data S12). For each panel, unexpected classes of cells were identified and integrated into the script. The original script identifying only expected phenotypes was compared to the updated script incorporating unexpected phenotypes, and differences in cell classification were only observed for MP1 (Fig. 7B). Such differences were seen exclusively in the TMA cores containing lymphoid tissue, wherein a portion (up to 16%) of CD4^+^ cells was reclassified as CD4^+^/CD8^+^ cells. All of these cells were located within groups of immune cells; none were isolated CD4^+^ cells (Fig. 7C).

Multimarker classification was also performed using qupath v0.2.0‐m9 to compare its novel multiplexed analysis approach (methods 1 and 2) to our current hierarchical method. Results of this analysis determined that cell classification in v0.2.0‐m9 intuitively enables multiclass cell labelling, rendering the cell detection order extraneous. Comparison of multiclass labelled cells (in v0.2.0‐m9) to cells classified by our hierarchical method (in v0.2.0‐m4) for MP1 found that, in the absence of hierarchical structuring, cells in tissues containing tertiary lymphocytic structures were likely to be further subcategorised based on relative proximity to CD20^+^ cells (Data S13). Using v0.2.0‐m9 method 1, significant differences in cell positivity were observed for CD3^−^/CD4^+^ and CD3^+^/CD8^+^ phenotypes in lymph node tissue (*P*‐values of 0.0173 and 0.0162, respectively). Method 2 introduced even greater differences across multiple cell phenotypes (CD20^+^, CD3^−^/CD8^+^, CD3^+^/CD8^+^, CD3^+^ and CK^+^; *P*‐values < 0.05) and tissue types, deriving from the over‐representation of CD20^+^ cells as well as the use of machine learning. Similar differences were observed for MP2; only this time, CD4^+^ cells were being further categorised by CD68 positivity. However, the percentage of CD4^+^/CD68^+^ cells was only statistically significant in tissue with high CD68 expression, spleen with method 1 and lymph node with method 2 (*P*‐values of 0.0212 and 0.0275, respectively). Since CD4 constitutes a biomarker in both panels, whose detection differed between v0.2.0‐m4 and v0.2.0‐m9, we compared the three multiplexed analyses of CD4 to the CD4 DAB data across all tissue types. Classification accuracy decreased from v0.2.0‐m4 (*r*_s_ = 0.958, *P* < 0.0001, bias = 0.07235 in MP1; *r*_s_ = 0.9209, *P* < 0.0001, bias = 1.907 in MP2) > v0.2.0‐m9 method 1 (*r*_s_ = 0.9308, *P* < 0.0001, bias = 5.492 in MP1; *r*_s_ = 0.9209, *P* < 0.0001, bias = 1.991 in MP2) > v0.2.0‐m9 method 2 (*r*_s_ = 0.9309, *P* < 0.0001, bias = 4.276 in MP1; *r*_s_ = 0.9118, *P* < 0.0001, bias = 1.493 in MP2).

### 3.7 Testing the validated workflow: from staining to digital image analysis

For completion of the study, the validated mIF protocols were performed on 10 full‐face CRC patient samples (five for MP1 and five for MP2). Validity of successful multispectral unmixing was assessed using the autofluorescence channel. Consequently, three sections were excluded from analysis due to levels of autofluorescence exceeding the spectral range used for autofluorescence subtraction, causing incomplete spectral unmixing. The v0.2.0‐m4 scripts were applied for classification of both expected and unexpected phenotypes. MP2‐stained sections exhibited no differences between scripts with or without unexpected classes, similarly to the TMA. Likewise with the TMA, MP1‐stained samples demonstrated a difference in phenotype results between the two scripts, with up to 5% of CD4^+^ cells being recategorised as CD4^+^/CD8^+^ cells within immune dense regions, thus highlighting the need for binary hierarchical classification.

## 4 Discussion

This study conclusively demonstrates the reliability and credibility of mIF in assessing immune biomarkers using the quantitative DIA qupath. mIF is unique in its ability to detect both the expression and geographic cellular distribution of immune biomarkers while preserving tissue architecture, contrarily to other techniques such as flow cytometry [17]. Herein, we present the first study to clearly define and demonstrate the technical limitations that exist for DIA of mIF tissue samples, more specifically, mIF using Opal fluorophores to detect co‐expressing immune biomarkers in the same cellular compartment.

For both of our mIF panels, all immunophenotypes were confirmed using singleplex DAB IHC and any steric hindrance or spectral crosstalk was ruled out using dropout controls. MP1 included three biomarkers (CD3, CD4 and CD8) that colocalised in the same cellular compartment of the same cell, whereas MP2 consisted of two biomarkers that co‐expressed in different cellular compartments (CD4 and FOXP3). Both panels included CD4 and CK, allowing panel design comparison. Using normal human tissue types, we identified the importance of the detection order in image analysis when assessing sites of densely populated co‐expressing immune cells, such as lymph node and tonsil. In these regions, cell detection was predisposed to overestimating whichever immune cell type was initially classified in the sequential binary classifier. This is because biomarkers that share very close subcellular locations can hinder antibody binding, signal amplification and digital detection of their co‐expressers by steric hindrance and masking effects [8, 17]. As a result, incompatible immunophenotypes were obtained when simultaneously classifying multiple biomarkers in tissue stained with MP1, but not with MP2. Incompatibility may arise as a result of overlapping cells in thick tissue sections or as a consequence of minimal focal planes in thin sections [18]. However, these factors were ruled out by the acceptable autofluorescent levels present in the validation TMA sections and the concordance observed for each biomarker between the paired singleplex DAB IHC and the mIF stains when binary phenotyping was used, in agreement with pathological review. In utilising this method, comparable CD4 and CK results were obtained in MP1 and MP2. These findings are in accordance with recent publications in this area, wherein binary phenotyping has been reported as being superior to multiple phenotyping in terms of sensitivity in detecting true unexpected dual expressions [17, 19]. Interestingly, inclusion of CD3 in the first panel permitted identification of a subpopulation of CD3‐positive, CD4/CD8 null immune cells that would otherwise not have been identified. This population of cells is well reported in other multiplexing technologies such as flow cytometry [19].

The importance of sequential, binary and overall hierarchical classification is reinforced by our v0.2.0‐m9 data. Although the classifiers were combined in a specific order, the cell classification in v0.2.0‐m9 was in fact nonhierarchical. To bypass this issue while the qupath milestone remains a work in progress, the additional classes can be reclassified into their respective true phenotypes using bespoke scripting, which we did not apply for the sole purpose of differentiating the effects of binary and multiple phenotyping. Moreover, our findings emphasise the need to understand the expected immunophenotypes when carrying out DIA. CD4/CD8 dual positivity, for instance, was accepted as true based on current literature [20]. CD4/CD68 double positivity, on the other hand, would require further investigation since this phenotype exists in the literature as a low CD68 expresser [21]. This would encompass reanalysing the DAB and MP2 data with appropriate thresholds for low‐ and high‐expressing CD68 cells. Furthermore, the controversial classes of cells additionally identified in v0.2.0‐m9 were more substantially present in method 2 using the random forest machine learning, a method that is highly accurate using limited training data but is liable to systematic errors when applied across large cohorts, and is thus apt to introducing bias data [22]. We therefore deduce thresholding to be a more accurate approach in the current study.

The determination of appropriate and definitive thresholds for each particular biomarker in the multiplex panels was enabled by performing DAB IHC in the initial steps of mIF optimisation [15]. Essentially, each target required wet‐lab optimisation for DAB IHC, singleplex IF and the full mIF assay on control tissues to guarantee reproducible digital results. Interestingly, the targets that were most affected by successive epitope retrieval when moving from DAB IHC to mIF have been reported as being sensitive to HIER prior to their use in mIF, a process that requires successive rounds of HIER to remove the previously bound antibody [15]. Among the methods of HIER, we noticed that alkaline‐based ER2 (pH 9.0) was prone to inducing high background signal compared to acidic ER1 (pH 6.0), which is supported elsewhere in the literature [23]. Furthermore, by means of DAB IHC we were able to discern the cause of the CD20‐CD8 crosstalk present in the germinal centres of the fluorescent images. These staining artefacts arose as a consequence of epitope instability in the multiplex. Decreasing epitope stability in sequential staining has been previously reported and can be mitigated by antibody positioning in the staining sequence [8]. However, in this study we demonstrate that placement in the optimal position may not be possible due to other biomarkers present in the panel, thus resulting in a trade‐off in accuracy which can be moderated using selective binary phenotyping [19].

The multiplex panels in the current study were developed using Opal reagents based on TSA methodology. During optimisation, we showed that the primary incubation times used for DAB IHC were identical for TSA‐based IF, thus confirming the use of TSA as a DAB equivalent [7]. TSA is most advantageous for its high sensitivity and specificity in detecting low‐expressing biomarkers, and in its ability to minimise the risk of antibody cross‐reactivity [5]. In our acquired images, we did not see any nonspecific background staining except for FOXP3 antibody in MP2, where red blood cells exhibited nonspecific FOXP3 reactivity. This phenomenon has been reported elsewhere and is in fact specific to the mIF assay, since no cross‐reactivity was observed in the singleplex DAB IHC [5, 24]. Furthermore, it has been shown in the literature that CK staining can be inconsistent between mIF batches compared to DAB IHC [15]. We were able to alleviate this problem and obtain CK mIF staining comparable to the DAB IHC by merely changing the Opal‐antibody pairing for CK, coupling it to the brightest Opal dye (Opal 480).

In addition to optimising the staining protocol, the use of multispectral unmixing aided the determination of positive cell detection. By unmixing the individual channels, clean images were produced, free from autofluorescence and crosstalk. Similarly to other studies, we found when utilising FFPE tissue the use of multispectral unmixing was most expedient over traditional IF image capture, because unlike fresh frozen tissue FFPE tissue is inherently autofluorescent [2]. By using the Opal MOTiF reagents in conjunction with the Vectra Polaris, we were able to quickly, reliably and reproducibly generate results from a digital mIF image in under 24 h from time of tissue sectioning. Even without MOTiF technology, the Opal methodology permits high‐throughput biomarker analysis [25]. Ideally, mIF would be carried out on fresh frozen tissue sections; however, tissue architecture is not as well preserved in fresh frozen tissue as FFPE [26]. Since most routinely processed and retrospectively collected tissue are FFPE, studies have been conducted to find an optimal long‐term fixative suitable for processing tissue for fluorescence [27]. So for mIF to be introduced into the clinical setting, robust automated protocols that can be independently validated are required. Formerly, manual mIF took 3 days at the bench, but now automated protocols can be ran overnight [5, 15]. While assay automation offers speed and reproducibility, errors may still arise, such as the appearance of a staining gradient across a slide [12]. However, this phenomenon was not observed in samples assessed for DIA in the current study, owing to the regular preventative maintenance and calibration of the autostainer as well as the stringent review of all slides considered for DIA during quality control procedures. Thus, with automated whole‐slide scanning and DIA, mIF is becoming more amenable in facilitating high‐throughput use, as demonstrated in the current study and previous studies [2, 5, 28]. Our further investigation into scanning parameters found that increasing magnification from 20× to 40× did not improve the delineation of individual cellular phenotypes when digitally assessing co‐expressing biomarkers in the same cellular compartment. In fact, difficulty in accurately phenotyping densely populated cells in fluorescent histological images has been reported elsewhere, thus supporting our findings and our decision to use quicker scanning times to produce images for high‐throughput use in this study [15, 19]. In future, the classical approach of watershed cell detection currently utilised in qupath may be superseded by use of deep learning‐based methods for nucleus segmentation, particularly when analysing regions of crowded cells [29]. A promising approach would involve incorporating StarDist 2D, a method that localises nuclei via star‐convex polygons, into qupath by scripting [10, 30].

An added benefit of using multispectral imaging and collecting in the autofluorescent channel was the determination of relative tissue thickness. We found that the predefined thresholds, based on initial DAB IHC and calibrated to the mIF validation assay, were dependent on very slight changes in tissue thickness. In essence, thresholds were significantly affected when the tissue section being assessed was cut either slightly thicker or thinner than expected. Natural variation in tissue thickness is expected and often seen in routine basic and special stains due to manual sectioning of the tissue. It is a recognised issue in manual IF assessment and one that is becoming increasingly apparent when developing artificial intelligence algorithms in histological images [31, 32]. Compared to brightfield image analysis, assessment of mIF is uniquely sensitive to very subtle changes in tissue thickness, which can either increase or decrease the potential availability of exposed epitopes to fluorophore binding [33]. This can result in underexposure or oversaturation of the fluorophore when imaged with the multispectral scanning protocol, which has been optimised for a particular tissue thickness. Therefore, the use of tissue sections with regular thin cuts is highly recommended for mIF assays [7, 31, 32].

Colour inconsistency is an issue in histological images that arises from pre‐analytical variables such as section thickness, which has become more palpable with the emergence of DIA. Image normalisation has been used with great effect in the digital assessment of brightfield images that display such colour inconsistency [34]. We attempted to apply image normalisation techniques to recalibrate the staining intensity of tissues that exhibited different thicknesses, but found it introduced digital artefacts and rendered the images not fit for purpose (data not shown). Although the inform software used for multispectral unmixing normalised each image to the reference channels of the synthetic library, it did not account for tissue thickness variation as only one autofluorescent spectrum was provided. A prospective approach to correcting for intersection thickness variability, which was not assessed in this study, would therefore involve using autofluorescent spectra captured from slides of varying thicknesses in combination with the synthetic Opal library. This would enable complete spectral unmixing and more accurate DIA, irrespective of the inherent autofluorescence of FFPE tissue.

## 5 Conclusion

This study not only demonstrates the importance of optimising the wet‐lab workflow for the production of quality mIF images, but also highlights the significance of standardising the DIA protocol before undertaking any large mIF studies. Detailed quality control of every step, from staining to analysis, is therefore necessary in ensuring accurate cellular profiling. Herein, we present a digital pathology workflow that allows for automated high‐throughput mIF and accurate immunophenotyping. Although there exist novel mIF platforms that offer much larger plex panels than the method used in this study, such as Akoya's CO‐Detection by indEXing (CODEX), Nanostring's Digital Spatial Profiling (DSP) and Ultivue's InSituPlex, these incur many limitations that do not permit cost‐ and time‐effective whole‐slide imaging. Equally, there are several dia software that are compatible with most mIF imaging modalities, including Indica Labs' HALO and Visiopharm's Oncotopix. However, unlike qupath, these proprietary software do not offer scripting functionalities, a feature considered essential to our study [35]. We also demonstrate the importance of minimising pre‐analytical variables for the assessment of the resultant digital image, including biomarker combinations of interest and tissue thickness. Such considerations permitted the creation of a reproducible pipeline for the quantitative assessment of mIF assays in FFPE tissue. Moreover, we found that pathologist supervision during the development of the DIA protocol was invaluable as certain immunophenotyping combinations being interrogated may have otherwise been overlooked [2]. To conclude, this study provides a robust, methodological guide for mIF validation for use in cancer immunology studies.

## Conflict of interest

Prof Phil Quirke has research funding with Roche, GeneFirst and Amgen, previous research funding from Halio, consultancy with Nordlai‐Adlyte and advisory boards with Merck, Amgen and Roche. Prof Manuel Salto‐Tellez is a senior scientific advisor to Philips Computational Pathology and Sonrai Analytics, and has received honoraria from Roche, AstraZeneca, Merck and GSK. These declarations of interest have no relationship with the submitted publication. All other authors declare no competing interests.

## Author contributions

AVP, SGC, VB, KM, MPH, SS, SDR, PQ, LC, ED, TSM, JAJ and MS‐T contributed to interpretation of data and writing of the manuscript. AVP, SGC, JAJ and MS‐T were involved in study conception and design. AVP, SGC, VB, SDR and LC contributed to data acquisition. AVP, SGC, KM and SS were involved in data analysis.

## Supporting information


**Data S1.** Optimised chromogenic singleplex IHC protocols for the biomarkers in MP1.

**Data S2.** List of antibodies used in the study.

**Data S3.** Spectra comparison of our measured study‐specific library and the readily available synthetic library.

**Data S4.** Drop‐out controls.

**Data S5.** The optimised MP2 protocol with the spectrum of the MOTiF Opals used.

**Data S6.** Comparison of 20x and 40x scanning magnifications after image analysis.

**Data S7.** MP2 validation results.

**Data S8.** Non‐specific CD8 staining in MP1.

**Data S9.** Insignificance of the detection order in digital assessment of MP2.

**Data S10.** Script used for multi‐marker classification of cells in MP1.

**Data S11.** Script used for multi‐marker classification of cells in MP2.

**Data S12.** Decision tree visually representing the script used for MP2 cell classification.

**Data S13.** Comparison of MP1 phenotype results obtained in QuPath v0.2.0‐m4 and v0.2.0‐m9 (method one and two).

## Data Availability

Data are held within the Northern Ireland Biobank and the stratified medicine consortium in colorectal cancer (S:CORT), and are available upon reasonable request.
