# Supplementary material for: A robust multiplex immunofluorescence and digital pathology workflow for the characterisation of the tumour immune microenvironment
Source: Mol Oncol. 2020 Sep 1;14(10):2384–402. doi: 10.1002/1878-0261.12764 (PMC7530793; doi:10.1002/1878-0261.12764)
Supplement: Supplementary file 1 — Data S1. Optimised chromogenic singleplex IHC protocols for the biomarkers in MP1. [file MOL2-14-2384-s001.docx]

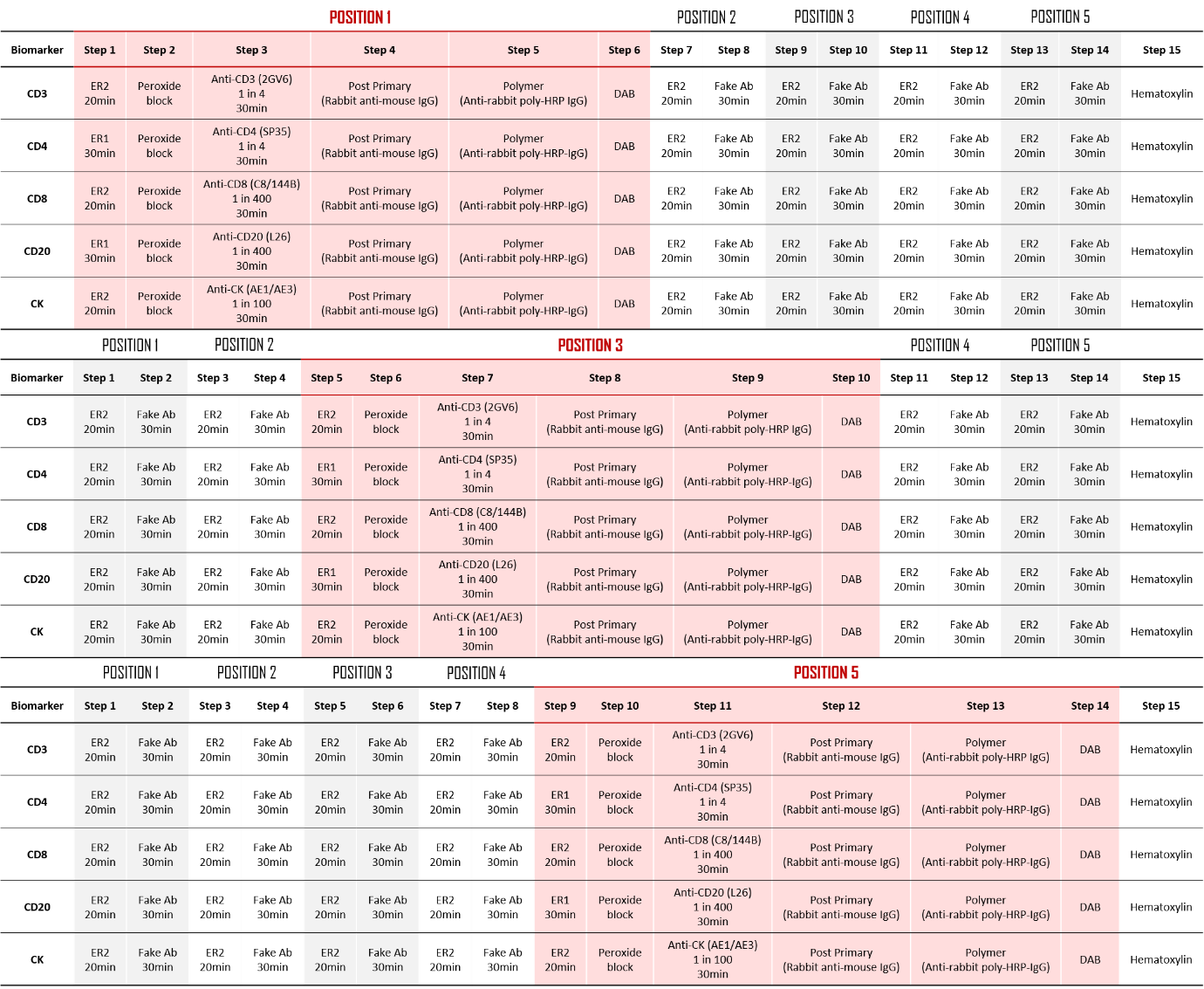


**Supplementary Data S1.** Optimised chromogenic singleplex IHC protocols for the biomarkers in MP1. Each singleplex (row) includes the appropriate number of epitope retrievals (ER) before and after antibody application so as to mimic a multiplex. All primary antibodies are either Mouse or Rabbit monoclonal antibodies. These were added in positions 1, 3 and 5 of the staining sequence, highlighted in red. With five biomarkers and three singeplexes per biomarker, there was a total of fifteen singleplex protocols. Fake Ab = Leica BOND wash buffer.
