# Supplementary material for: A robust multiplex immunofluorescence and digital pathology workflow for the characterisation of the tumour immune microenvironment
Source: Mol Oncol. 2020 Sep 1;14(10):2384–402. doi: 10.1002/1878-0261.12764 (PMC7530793; doi:10.1002/1878-0261.12764)
Supplement: Supplementary file 2 — Data S2. List of antibodies used in the study. [file MOL2-14-2384-s002.docx]

| **Biomarker** | **Antibody** | **Catalogue Number** | **Clone** | **Source** | **Immunostainer** | **Epitope Retrieval** | **Dilution** | **Incubation** | **Detection Chemistry** | **Positive Control** |
| --- | --- | --- | --- | --- | --- | --- | --- | --- | --- | --- |
| CD3 | Anti-CD3 | 5278422001 | 2GV6 | Ventana | Leica BOND RX | ER2 for 20 minutes | 1 in 4 | 30 minutes at 37°C | BOND Polymer Refine Detection | Tonsil |
| CD4 | Anti-CD4 | 5552737001 | SP35 | Ventana | Leica BOND RX | ER1 for 30 minutes | 1 in 4 | 30 minutes at 37°C | BOND Polymer Refine Detection | Tonsil |
| CD8 | Anti-CD8 | M7103 | C8/144B | Dako | Leica BOND RX | ER2 for 20 minutes | 1 in 400 | 30 minutes at 37°C | BOND Polymer Refine Detection | Tonsil |
| CD20 | Anti-CD20 | M0755 | L26 | Dako | Leica BOND RX | ER1 for 30 minutes | 1 in 400 | 30 minutes at 37°C | BOND Polymer Refine Detection | Tonsil |
| CK | Anti-CK | M3515 | AE1/AE3 | Dako | Leica BOND RX | ER2 for 20 minutes | 1 in 100 | 30 minutes at 37°C | BOND Polymer Refine Detection | Tonsil |
| CD3 | Anti-CD3 | 5278422001 | 2GV6 | Ventana | Leica BOND RX | ER1 for 30 minutes | 1 in 6 | 30 minutes at 37°C | Opal Polaris 7-Color Automation IHC | Tonsil |
| CD4 | Anti-CD4 | 5552737001 | SP35 | Ventana | Leica BOND RX | ER1 for 30 minutes | 1 in 4 | 30 minutes at 37°C | Opal Polaris 7-Color Automation IHC | Tonsil |
| CD8 | Anti-CD8 | M7103 | C8/144B | Dako | Leica BOND RX | ER1 for 30 minutes | 1 in 400 | 30 minutes at 37°C | Opal Polaris 7-Color Automation IHC | Tonsil |
| CD20 | Anti-CD20 | M0755 | L26 | Dako | Leica BOND RX | ER1 for 30 minutes | 1 in 400 | 30 minutes at 37°C | Opal Polaris 7-Color Automation IHC | Tonsil |
| CK | Anti-CK | M3515 | AE1/AE3 | Dako | Leica BOND RX | ER1 for 30 minutes | 1 in 100 | 30 minutes at 37°C | Opal Polaris 7-Color Automation IHC | Tonsil |
| CD68 | Anti-CD68 | NCL-L-CD68 | 514H12 | Novocastra | Leica BOND RX | ER1 for 30 minutes | 1 in 200 | 30 minutes at 37°C | Opal Polaris 7-Color Automation IHC | Tonsil |
| FOXP3 | Anti-FOXP3 | LS-C210349 | SP97 | LSBio | Leica BOND RX | ER2 for 20 minutes | 1 in 200 | 30 minutes at 37°C | Opal Polaris 7-Color Automation IHC | Tonsil |

**Supplementary Data S2:** List of antibodies used in the study. Antibody conditions determined in chromogenic singleplex IHC (first five rows) were applied to the fluorescent assays and adjusted throughout mIF optimisation. The antibody conditions used in the final optimised MP1 and MP2 IF protocols are depicted (last seven rows).
