# Supplementary material for: A robust multiplex immunofluorescence and digital pathology workflow for the characterisation of the tumour immune microenvironment
Source: Mol Oncol. 2020 Sep 1;14(10):2384–402. doi: 10.1002/1878-0261.12764 (PMC7530793; doi:10.1002/1878-0261.12764)
Supplement: Supplementary file 3 — Data S3. Spectra comparison of our measured study‐specific library and the readily available synthetic library. [file MOL2-14-2384-s003.docx]

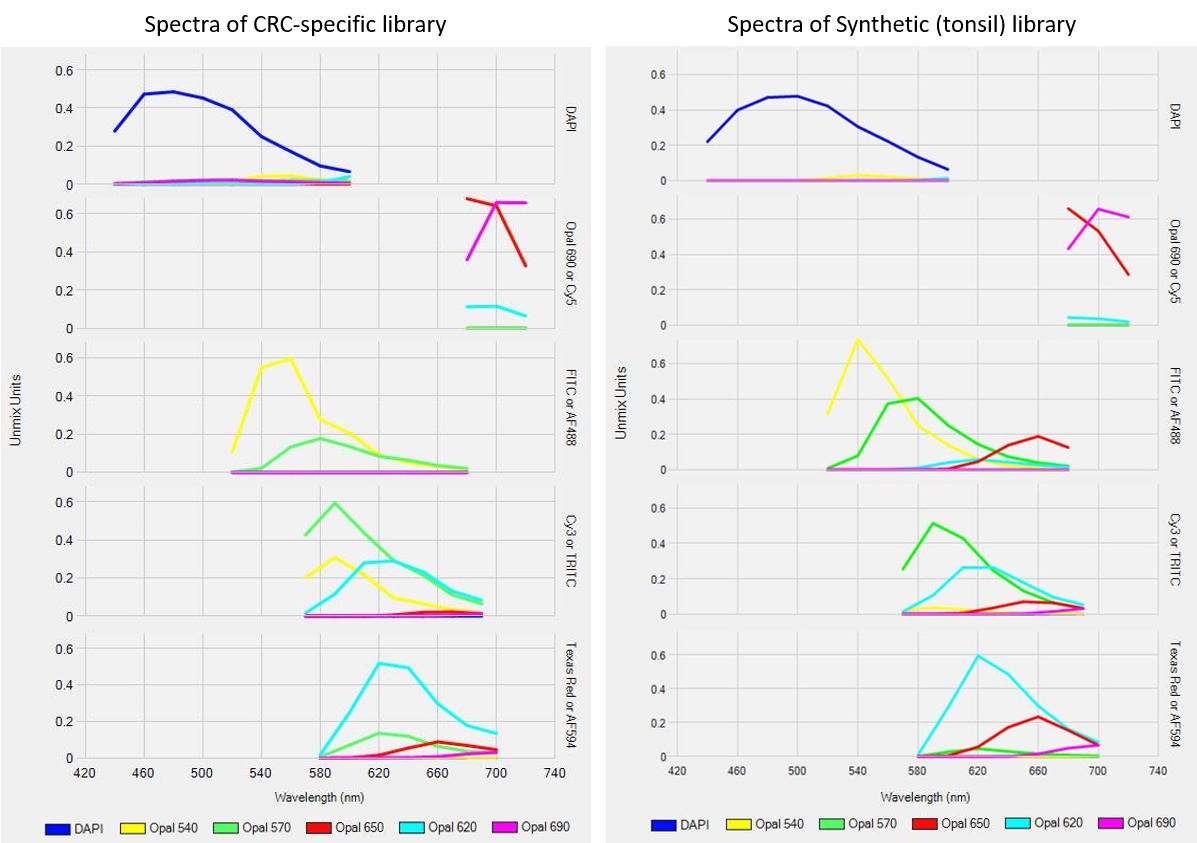


**Supplementary Data S3.** Spectra comparison of our measured study-specific library (left) and the readily available synthetic library (right). For the study-specific library, n = 7 CRC sections were used to create library slides and each fluorophore spectrum was extracted using inForm automated tools in the ‘Build Libraries’ tab. Each row corresponds to one of the five epi-fluorescence channels in which the fluorophore peaks emit: DAPI, Opals 650 and 690 emit in the Cy5 channel, Opal 540 in FITC, Opal 570 in Cy3, and Opal 620 in Texas Red. Note that these are the Opals that were used in the first proposed MP1 protocol and not the final optimised protocol.
