# Supplementary material for: A robust multiplex immunofluorescence and digital pathology workflow for the characterisation of the tumour immune microenvironment
Source: Mol Oncol. 2020 Sep 1;14(10):2384–402. doi: 10.1002/1878-0261.12764 (PMC7530793; doi:10.1002/1878-0261.12764)
Supplement: Supplementary file 4 — Data S4. Drop‐out controls. [file MOL2-14-2384-s004.docx]

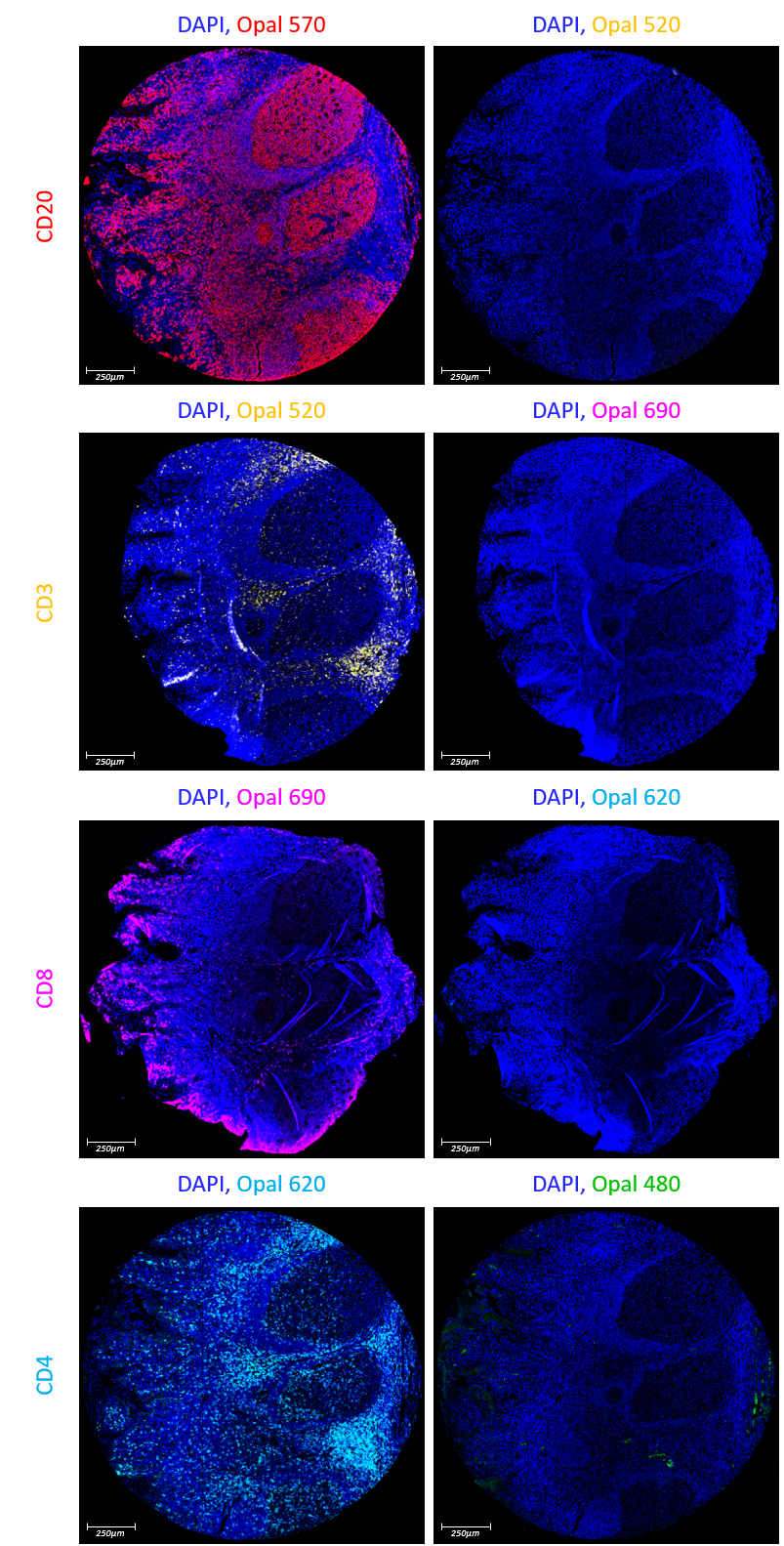


**Supplementary Data S4.** Drop-out controls determine whether previously bound antibodies have been adequately removed to prevent signal crosstalk. These were created using the final optimised MP1 protocol order (CD20 > CD3 > CD8 > CD4 > CK) and by running four individual staining protocols on tonsil tissue (n = 4). The first drop-out control (first row) consisted of running a complete first sequence (CD20 antibody, HRP polymer, Opal 570, ER1) then a second sequence without primary antibody (no CD3 antibody, but with HRP polymer, Opal 520 and ER1). The second control (second row) consisted of a complete second sequence and a partial third sequence (no CD8 antibody), and so forth. Results confirmed effective stripping after each antibody sequence i.e. no staining is seen in the channel without a primary antibody (right column). Images are viewed at 10x magnification (scale bar = 250 µm).
