# Supplementary material for: A robust multiplex immunofluorescence and digital pathology workflow for the characterisation of the tumour immune microenvironment
Source: Mol Oncol. 2020 Sep 1;14(10):2384–402. doi: 10.1002/1878-0261.12764 (PMC7530793; doi:10.1002/1878-0261.12764)
Supplement: Supplementary file 5 — Data S5. The optimised MP2 protocol with the spectrum of the MOTiF Opals used. [file MOL2-14-2384-s005.docx]

**
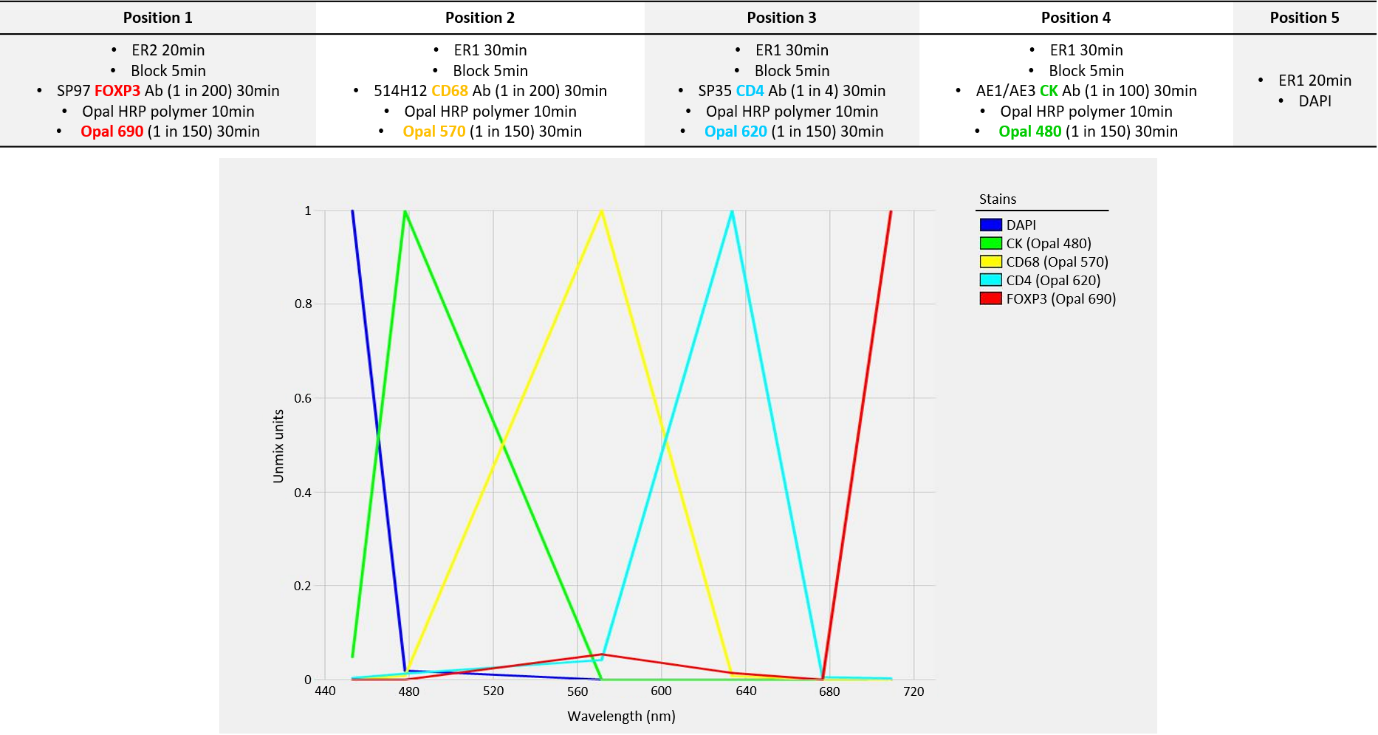
**

**Supplementary Data S5.** The optimised MP2 protocol with the spectrum of the MOTiF Opals used below. No overlap is seen between the emission peaks. Achieving the final optimised MP2 protocol required the development of two protocols and the use of n = 7 tissue sections.
