# Supplementary material for: A robust multiplex immunofluorescence and digital pathology workflow for the characterisation of the tumour immune microenvironment
Source: Mol Oncol. 2020 Sep 1;14(10):2384–402. doi: 10.1002/1878-0261.12764 (PMC7530793; doi:10.1002/1878-0261.12764)
Supplement: Supplementary file 6 — Data S6. Comparison of 20x and 40x scanning magnifications after image analysis. [file MOL2-14-2384-s006.docx]

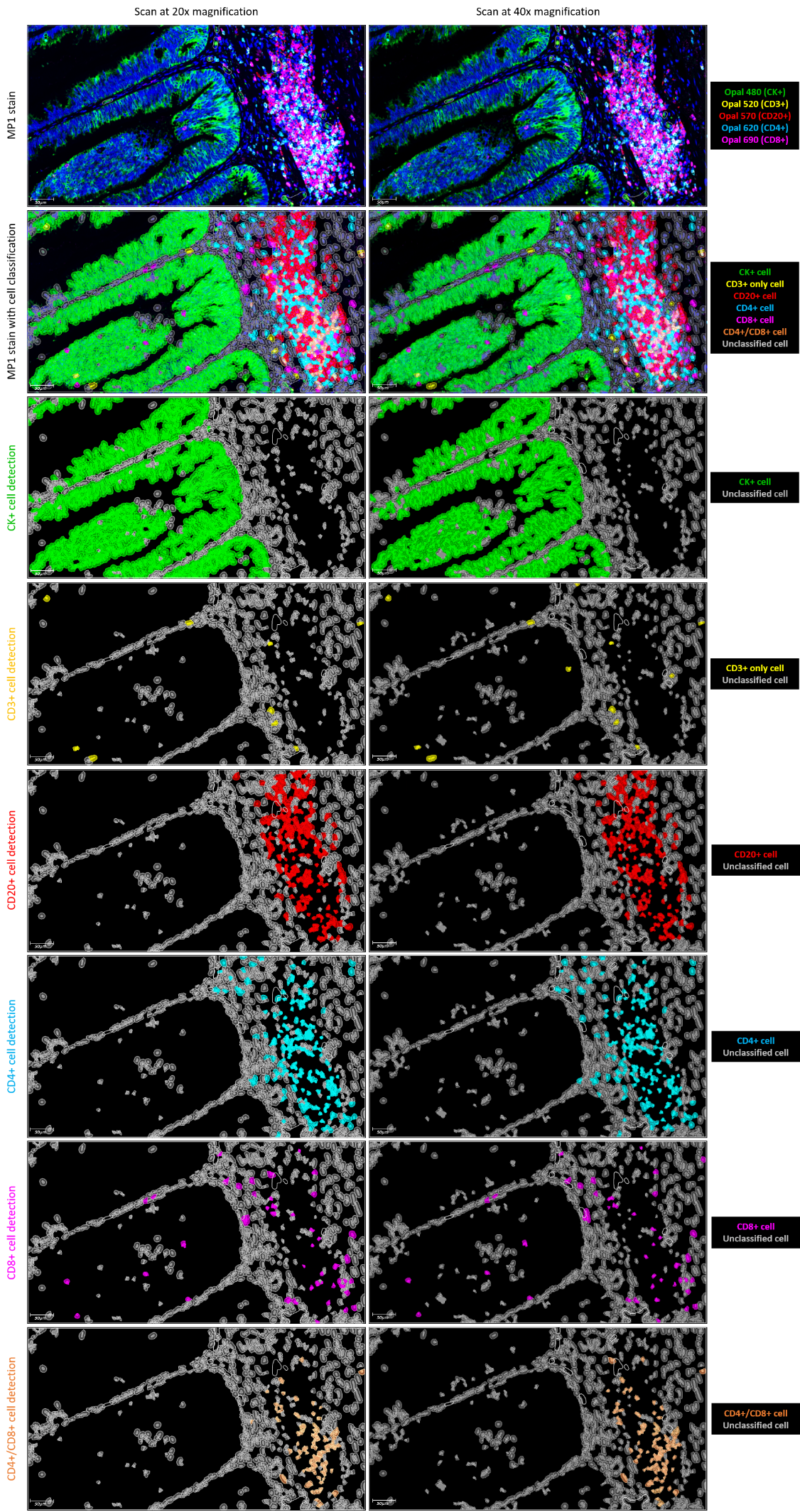


**Supplementary Data S6.** Comparison of 20x and 40x scanning magnifications after image analysis. The same region of the full-face CRC section that was presented in Figure 4A is shown here at 20x magnification (scale bar = 50 µm). Cell classification has been applied to the 20x and 40x scanned images (second row) and the results are almost identical (<1% difference in cell counts), irrespective of their scanning protocol. Rows 3-8 illustrate the individual biomarkers. Unclassified cells are displayed in grey.
