# Supplementary material for: A robust multiplex immunofluorescence and digital pathology workflow for the characterisation of the tumour immune microenvironment
Source: Mol Oncol. 2020 Sep 1;14(10):2384–402. doi: 10.1002/1878-0261.12764 (PMC7530793; doi:10.1002/1878-0261.12764)
Supplement: Supplementary file 7 — Data S7. MP2 validation results. [file MOL2-14-2384-s007.docx]

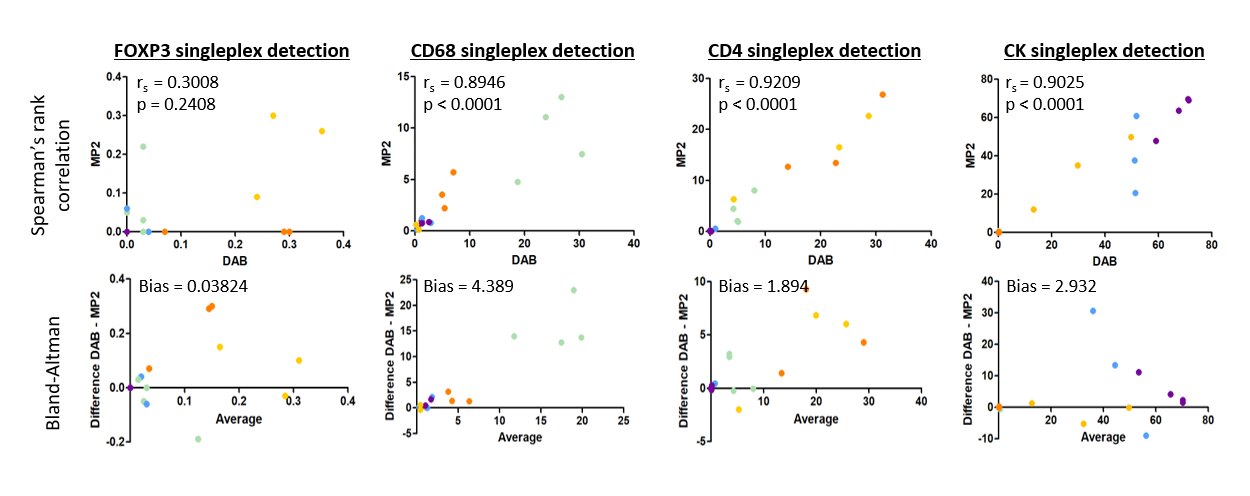


**Supplementary Data S7.** MP2 validation results. Spearman’s rank correlation graphs (top) and Bland-Altman plots (bottom) illustrating the relationship and agreement between DAB detection (from TMAs 6, 7, 8, 9) and singleplex IF detection (from TMA 5), for each biomarker in MP2. The same cores (n = 17) across all the TMAs were used for analysis of the biomarkers. All correlations are significantly strong except for FOXP3, and all biases are within the limits of agreement.
