# Supplementary material for: A robust multiplex immunofluorescence and digital pathology workflow for the characterisation of the tumour immune microenvironment
Source: Mol Oncol. 2020 Sep 1;14(10):2384–402. doi: 10.1002/1878-0261.12764 (PMC7530793; doi:10.1002/1878-0261.12764)
Supplement: Supplementary file 8 — Data S8. Non‐specific CD8 staining in MP1. [file MOL2-14-2384-s008.docx]

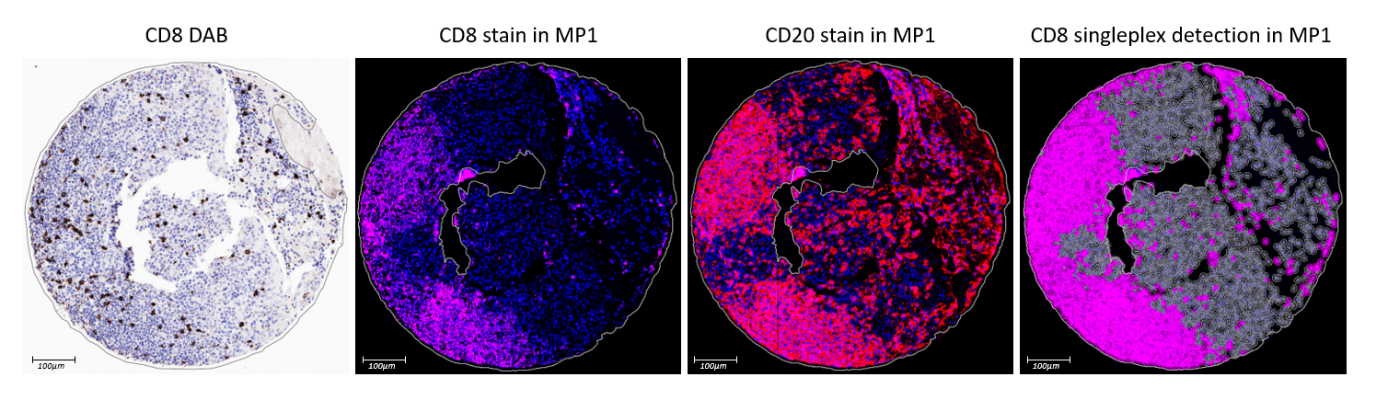


**Supplementary Data S8.** Non-specific CD8 staining in MP1. The left image is a tonsil core in TMA section 3 stained with CD8 DAB IHC. The right three images are of the same tonsil core in TMA section 4 stained with MP1 IF, but showing different channels (from left to right): CD8 channel, CD20 channel, and CD8 channel with overlaying cell detection (CD8+ cells in magenta and negative cells in grey). Due to CD8 antibody positioning in the multiplex, CD8 epitope stability is reduced and staining is rendered non-specific, thus staining true CD20+ lymphoid follicles as false CD8+. Images are displayed at 10x magnification (scale bar = 100 µm).
