# Supplementary material for: A robust multiplex immunofluorescence and digital pathology workflow for the characterisation of the tumour immune microenvironment
Source: Mol Oncol. 2020 Sep 1;14(10):2384–402. doi: 10.1002/1878-0261.12764 (PMC7530793; doi:10.1002/1878-0261.12764)
Supplement: Supplementary file 9 — Data S9. Insignificance of the detection order in digital assessment of MP2. [file MOL2-14-2384-s009.docx]

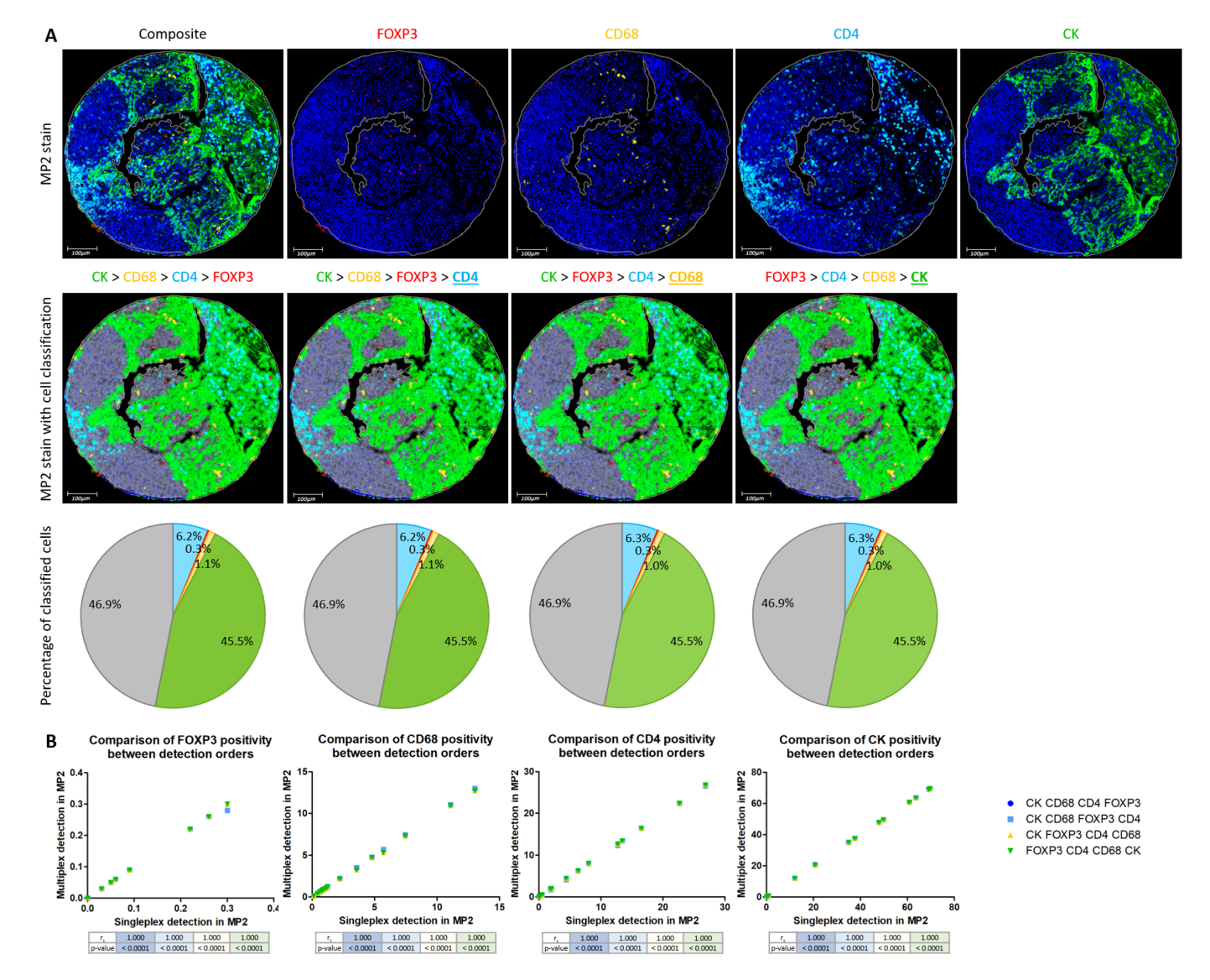


**Supplementary Data S9.** Insignificance of the detection order in digital assessment of MP2. (A) Example images of tonsil core 1 stained with MP2 protocol, seen at 10x magnification (scale bar = 100 µm). Top row shows the original stains: a composite image followed by an image for each individual marker. Middle row displays the same composite image with four different detection orders applied. From one order to the next, the biomarker that has been modified is underlined. Bottom row presents the phenotypes identified by each detection order as a percentage of the classified cells. Pie chart colours are in accordance with cell classification colours above. Unclassified cells are indicated in grey. All detection orders produce the same classification results visually. (B) Scatter graphs showing the correlation between each multiplex detection order and the singleplex detection of MP2 TMA 5, for each biomarker. The same cores (n = 17) of MP2 TMA 5 were used for analysis of the biomarkers. Statistical significance was measured by Spearman’s rank correlation coefficient. Again, all detection orders generate the same data, strongly correlated to the singleplex detection data (r_s_ = 1, p < 0.0001).
