# Supplementary material for: A robust multiplex immunofluorescence and digital pathology workflow for the characterisation of the tumour immune microenvironment
Source: Mol Oncol. 2020 Sep 1;14(10):2384–402. doi: 10.1002/1878-0261.12764 (PMC7530793; doi:10.1002/1878-0261.12764)
Supplement: Supplementary file 10 — Data S10. Script used for multi‐marker classification of cells in MP1. [file MOL2-14-2384-s010.docx]

import static qupath.lib.gui.scripting.QPEx.*

import qupath.lib.objects.PathObjects

import qupath.lib.objects.classes.PathClassFactory

import qupath.lib.objects.classes.PathClassTools

measurement1 = "Cell: Opal 480 mean" //CK

measurement2 = "Nucleus: Opal 620 mean" //CD4

measurement3 = "Nucleus: Opal 520 mean" //CD3

measurement4 = "Nucleus: Opal 570 mean" //CD20

measurement5 = "Nucleus: Opal 690 mean" //CD8

CKPos = getPathClass("Tumor")

CKNeg = getPathClass("Stroma")

CD3Pos = getPathClass("Immune cells")

CD3Pos_TIL = getPathClass("TIL")

CD8Pos = getPathClass("CD8+")

CD8Pos_TIL = getPathClass("CD8+ TIL")

CD4Pos = getPathClass("CD4+")

CD4Pos_TIL = getPathClass("CD4+ TIL")

CD20Pos = getPathClass("CD20+")

CD20Pos_TIL = getPathClass("CD20+ TIL")

CD8Pos_CD3Neg = getPathClass("CD3-/CD8+")

CD4Pos_CD3Neg = getPathClass("CD3-/CD4+")

CD8Pos_CD3Neg_TIL = getPathClass("CD3-/CD8+ TIL")

CD4Pos_CD3Neg_TIL = getPathClass("CD3-/CD4+ TIL")

CD4Pos_CD8Pos = getPathClass("CD4+/CD8+")

CD4Pos_CD8Pos_CD3Neg = getPathClass("CD3-/CD4+/CD8+")

CD4Pos_CD8Pos_TIL = getPathClass("CD4+/CD8+ TIL")

CD4Pos_CD8Pos_CD3Neg_TIL = getPathClass("CD3-/CD4+/CD8+ TIL")

//classify tumor and stroma

selectObjects {it.isDetection()}

for (detection in getSelectedObjects()) {

m1 = measurement(detection, measurement1)

if ( m1 > 0.4 )

detection.setPathClass(CKPos)

else

detection.setPathClass(CKNeg)

}

fireHierarchyUpdate()

//classify CD4 in tumor

selectObjects { p -> p.getPathClass() == getPathClass("Tumor")}

for (detection in getSelectedObjects()) {

m2 = measurement(detection, measurement2)

if ( m2 > 0.55 )

detection.setPathClass(CD4Pos_TIL)

else

detection.setPathClass(CKPos)

}

fireHierarchyUpdate()

//classify CD4 in stroma

selectObjects { p -> p.getPathClass() == getPathClass("Stroma")}

for (detection in getSelectedObjects()) {

m2 = measurement(detection, measurement2)

if ( m2 > 0.55 )

detection.setPathClass(CD4Pos)

else

detection.setPathClass(CKNeg)

}

fireHierarchyUpdate()

//classify CD3 in tumor

selectObjects { p -> p.getPathClass() == getPathClass("Tumor")}

for (detection in getSelectedObjects()) {

m3 = measurement(detection, measurement3)

if ( m3 > 0.1 )

detection.setPathClass(CD3Pos_TIL)

else

detection.setPathClass(CKPos)

}

fireHierarchyUpdate()

//classify CD3 in stroma

selectObjects { p -> p.getPathClass() == getPathClass("Stroma")}

for (detection in getSelectedObjects()) {

m3 = measurement(detection, measurement3)

if ( m3 > 0.1 )

detection.setPathClass(CD3Pos)

else

detection.setPathClass(CKNeg)

}

fireHierarchyUpdate()

//classify CD20 in tumor

selectObjects { p -> p.getPathClass() == getPathClass("Tumor")}

for (detection in getSelectedObjects()) {

m4 = measurement(detection, measurement4)

if ( m4 > 0.65 )

detection.setPathClass(CD20Pos_TIL)

else

detection.setPathClass(CKPos)

}

fireHierarchyUpdate()

//classify CD20 in stroma

selectObjects { p -> p.getPathClass() == getPathClass("Stroma")}

for (detection in getSelectedObjects()) {

m4 = measurement(detection, measurement4)

if ( m4 > 0.65 )

detection.setPathClass(CD20Pos)

else

detection.setPathClass(CKNeg)

}

fireHierarchyUpdate()

//classify CD8 in tumor

selectObjects { p -> p.getPathClass() == getPathClass("Tumor")}

for (detection in getSelectedObjects()) {

m5 = measurement(detection, measurement5)

if ( m5 > 1.5 )

detection.setPathClass(CD8Pos_CD3Neg_TIL)

else

detection.setPathClass(CKPos)

}

fireHierarchyUpdate()

//classify CD8 in stroma

selectObjects { p -> p.getPathClass() == getPathClass("Stroma")}

for (detection in getSelectedObjects()) {

m5 = measurement(detection, measurement5)

if ( m5 > 1.5 )

detection.setPathClass(CD8Pos_CD3Neg)

else

detection.setPathClass(CKNeg)

}

fireHierarchyUpdate()

//classify CD4 and CD8 as dual positive for CD3

//classify CD3 in tumor CD4

selectObjects { p -> p.getPathClass() == getPathClass("CD4+ TIL")}

for (detection in getSelectedObjects()) {

m3 = measurement(detection, measurement3)

if ( m3 > 0.1 )

detection.setPathClass(CD4Pos_TIL)

else

detection.setPathClass(CD4Pos_CD3Neg_TIL)

}

fireHierarchyUpdate()

//classify CD3 in stroma CD4

selectObjects { p -> p.getPathClass() == getPathClass("CD4+")}

for (detection in getSelectedObjects()) {

m3 = measurement(detection, measurement3)

if ( m3 > 0.1 )

detection.setPathClass(CD4Pos)

else

detection.setPathClass(CD4Pos_CD3Neg)

}

fireHierarchyUpdate()

//classify CD8 in tumor CD3

selectObjects { p -> p.getPathClass() == getPathClass("TIL")}

for (detection in getSelectedObjects()) {

m5 = measurement(detection, measurement5)

if ( m5 > 1.5 )

detection.setPathClass(CD8Pos_TIL)

else

detection.setPathClass(CD3Pos_TIL)

}

fireHierarchyUpdate()

//classify CD8 in stroma CD3

selectObjects { p -> p.getPathClass() == getPathClass("Immune cells")}

for (detection in getSelectedObjects()) {

m5 = measurement(detection, measurement5)

if ( m5 > 1.5 )

detection.setPathClass(CD8Pos)

else

detection.setPathClass(CD3Pos)

}

fireHierarchyUpdate()

//classify CD4 and CD8 as dual positive CD4/CD8

//classify CD8 in tumor CD4

selectObjects { p -> p.getPathClass() == getPathClass("CD4+ TIL")}

for (detection in getSelectedObjects()) {

m5 = measurement(detection, measurement5)

if ( m5 > 1.5 )

detection.setPathClass(CD4Pos_CD8Pos_TIL)

else

detection.setPathClass(CD4Pos_TIL)

}

fireHierarchyUpdate()

//classify CD8 in tumor CD3-/CD4

selectObjects { p -> p.getPathClass() == getPathClass("CD3-/CD4+ TIL")}

for (detection in getSelectedObjects()) {

m5 = measurement(detection, measurement5)

if ( m5 > 1.5 )

detection.setPathClass(CD4Pos_CD8Pos_CD3Neg_TIL)

else

detection.setPathClass(CD4Pos_CD3Neg_TIL)

}

fireHierarchyUpdate()

//classify CD8 in stroma CD4

selectObjects { p -> p.getPathClass() == getPathClass("CD4+")}

for (detection in getSelectedObjects()) {

m5 = measurement(detection, measurement5)

if ( m5 > 1.5 )

detection.setPathClass(CD4Pos_CD8Pos)

else

detection.setPathClass(CD4Pos)

}

fireHierarchyUpdate()

//classify CD8 in stroma CD3-/CD4

selectObjects { p -> p.getPathClass() == getPathClass("CD3-/CD4+")}

for (detection in getSelectedObjects()) {

m5 = measurement(detection, measurement5)

if ( m5 > 1.5 )

detection.setPathClass(CD4Pos_CD8Pos_CD3Neg)

else

detection.setPathClass(CD4Pos_CD3Neg)

}

fireHierarchyUpdate()

**Supplementary Data S10.** Script used for multi-marker classification of cells in MP1. Lines 174-217 permit further subtyping of T lymphocytes into the unexpected dual positive class CD4+/CD8+.
