# Supplementary material for: A robust multiplex immunofluorescence and digital pathology workflow for the characterisation of the tumour immune microenvironment
Source: Mol Oncol. 2020 Sep 1;14(10):2384–402. doi: 10.1002/1878-0261.12764 (PMC7530793; doi:10.1002/1878-0261.12764)
Supplement: Supplementary file 11 — Data S11. Script used for multi‐marker classification of cells in MP2. [file MOL2-14-2384-s011.docx]

import static qupath.lib.gui.scripting.QPEx.*

import qupath.lib.objects.PathObjects

import qupath.lib.objects.classes.PathClassFactory

import qupath.lib.objects.classes.PathClassTools

measurement1 = "Cell: Opal 480 mean" //CK

measurement2 = "Nucleus: Opal 570 mean" //CD68

measurement3 = "Nucleus: Opal 690 mean" //FOXP3

measurement4 = "Nucleus: Opal 620 mean" //CD4

CKPos = getPathClass("Tumor")

CKNeg = getPathClass("Stroma")

CD68Pos = getPathClass("Immune cells")

CD68Pos_TIL = getPathClass("TIL")

CD4Pos = getPathClass("CD4+")

CD4Pos_TIL = getPathClass("CD4+ TIL")

FOXP3Pos = getPathClass("FOXP3+")

FOXP3Pos_TIL = getPathClass("FOXP3+ TIL")

FOXP3Pos_CD4Neg = getPathClass("CD4-/FOXP3+")

FOXP3Pos_CD4Neg_TIL = getPathClass("CD4-/FOXP3+ TIL")

CD68Pos_FOXP3Pos = getPathClass("CD68+/FOXP3+")

CD68Pos_FOXP3Pos_TIL = getPathClass("CD68+/FOXP3+ TIL")

//classify tumor and stroma

selectObjects {it.isDetection()}

for (detection in getSelectedObjects()) {

m1 = measurement(detection, measurement1)

if ( m1 > 0.4 )

detection.setPathClass(CKPos)

else

detection.setPathClass(CKNeg)

}

fireHierarchyUpdate()

//classify FOXP3 in tumor

selectObjects { p -> p.getPathClass() == getPathClass("Tumor")}

for (detection in getSelectedObjects()) {

m3 = measurement(detection, measurement3)

if ( m3 > 2 )

detection.setPathClass(FOXP3Pos_TIL)

else

detection.setPathClass(CKPos)

}

fireHierarchyUpdate()

//classify FOXP3 in stroma

selectObjects { p -> p.getPathClass() == getPathClass("Stroma")}

for (detection in getSelectedObjects()) {

m3 = measurement(detection, measurement3)

if ( m3 > 2 )

detection.setPathClass(FOXP3Pos)

else

detection.setPathClass(CKNeg)

}

fireHierarchyUpdate()

//classify CD4 in tumor

selectObjects { p -> p.getPathClass() == getPathClass("Tumor")}

for (detection in getSelectedObjects()) {

m4 = measurement(detection, measurement4)

if ( m4 > 2 )

detection.setPathClass(CD4Pos_TIL)

else

detection.setPathClass(CKPos)

}

fireHierarchyUpdate()

//classify CD4 in stroma

selectObjects { p -> p.getPathClass() == getPathClass("Stroma")}

for (detection in getSelectedObjects()) {

m4 = measurement(detection, measurement4)

if ( m4 > 2 )

detection.setPathClass(CD4Pos)

else

detection.setPathClass(CKNeg)

}

fireHierarchyUpdate()

//classify CD68 in tumor

selectObjects { p -> p.getPathClass() == getPathClass("Tumor")}

for (detection in getSelectedObjects()) {

m2 = measurement(detection, measurement2)

if ( m2 > 0.5 )

detection.setPathClass(CD68Pos_TIL)

else

detection.setPathClass(CKPos)

}

fireHierarchyUpdate()

//classify CD68 in stroma

selectObjects { p -> p.getPathClass() == getPathClass("Stroma")}

for (detection in getSelectedObjects()) {

m2 = measurement(detection, measurement2)

if ( m2 > 0.5 )

detection.setPathClass(CD68Pos)

else

detection.setPathClass(CKNeg)

}

fireHierarchyUpdate()

//classify FOXP3 as dual positive for CD4

//tumour

selectObjects { p -> p.getPathClass() == getPathClass("FOXP3+ TIL")}

for (detection in getSelectedObjects()) {

m4 = measurement(detection, measurement4)

if ( m4 > 2 )

detection.setPathClass(FOXP3Pos_TIL)

else

detection.setPathClass(FOXP3Pos_CD4Neg_TIL)

}

fireHierarchyUpdate()

//stroma

selectObjects { p -> p.getPathClass() == getPathClass("FOXP3+")}

for (detection in getSelectedObjects()) {

m4 = measurement(detection, measurement4)

if ( m4 > 2 )

detection.setPathClass(FOXP3Pos)

else

detection.setPathClass(FOXP3Pos_CD4Neg)

}

fireHierarchyUpdate()

**Supplementary Data S11.** Script used for multi-marker classification of cells in MP2.
