# Supplementary material for: A robust multiplex immunofluorescence and digital pathology workflow for the characterisation of the tumour immune microenvironment
Source: Mol Oncol. 2020 Sep 1;14(10):2384–402. doi: 10.1002/1878-0261.12764 (PMC7530793; doi:10.1002/1878-0261.12764)
Supplement: Supplementary file 12 — Data S12. Decision tree visually representing the script used for MP2 cell classification. [file MOL2-14-2384-s012.docx]

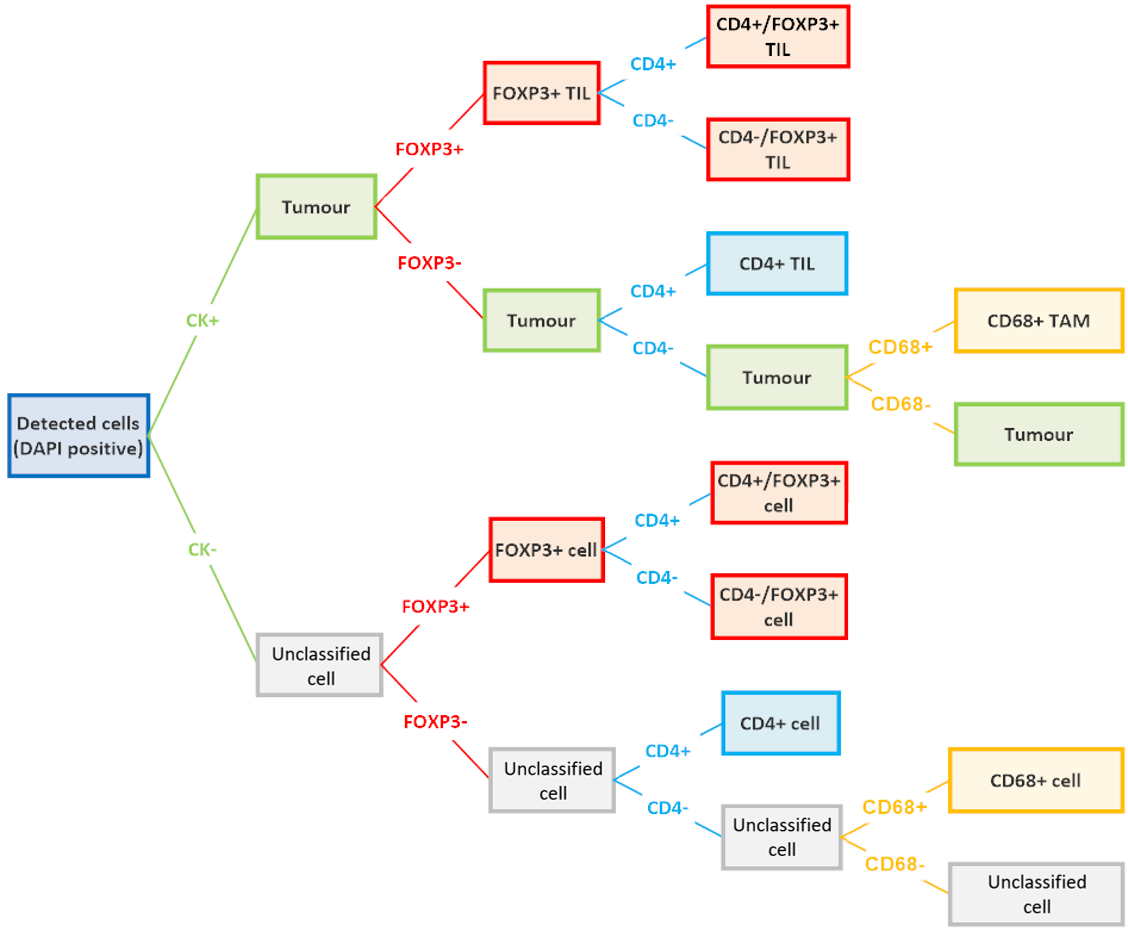


**Supplementary Data S12.** Decision tree visually representing the script used for MP2 cell classification. TIL = Tumour infiltrating lymphocyte; TAM = Tumour associated macrophage.
