# Supplementary material for: A robust multiplex immunofluorescence and digital pathology workflow for the characterisation of the tumour immune microenvironment
Source: Mol Oncol. 2020 Sep 1;14(10):2384–402. doi: 10.1002/1878-0261.12764 (PMC7530793; doi:10.1002/1878-0261.12764)
Supplement: Supplementary file 13 — Data S13. Comparison of MP1 phenotype results obtained in QuPath v0.2.0‐m4 and v0.2.0‐m9 (method one and two). [file MOL2-14-2384-s013.docx]

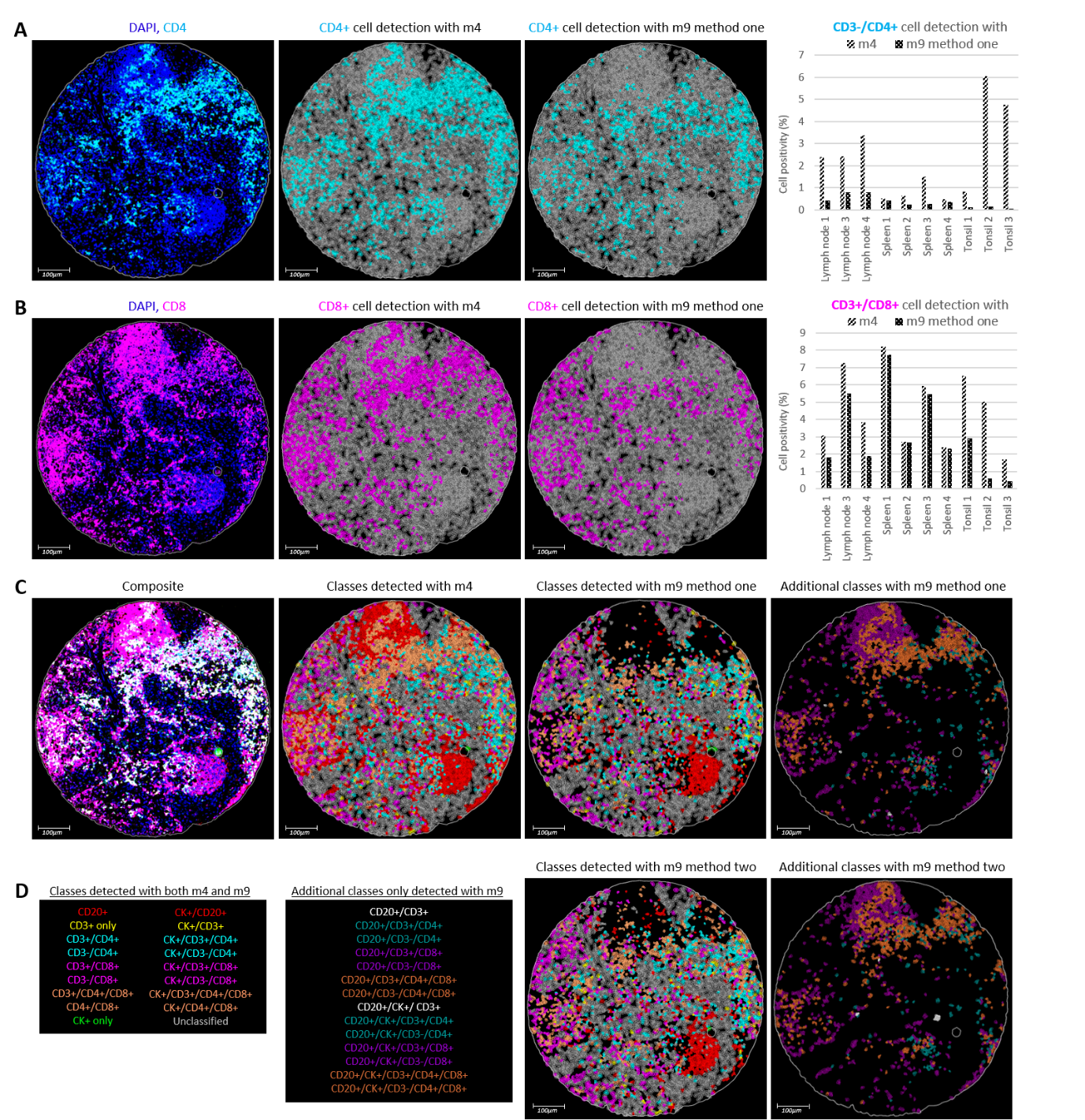


**Supplementary Data S13.** Comparison of MP1 phenotype results obtained in QuPath v0.2.0-m4 and v0.2.0-m9 (method one and two). Images are of lymph node 3 core, seen here at 10x magnification (scale bar = 100 µm). Statistical significance was determined by a paired t-test. (A) Difference in CD4 positive cell detection. From left to right: Original MP1 stain showing only DAPI and CD4 staining, followed by the same image with CD4+ detection applied in v0.2.0-m4 and v0.2.0-m9 method one. CD4+ cells are seen in cyan and negative cells in grey. Bar graph illustrates the percentage of CD3-/CD4+ cells detected in the lymphoid cores (n = 10) using v0.2.0-m4 and v0.2.0-m9 method one. The only significant difference in cell positivity is in lymph node tissue (p=0.0173). (B) Difference in CD8 positive cell detection. From left to right: Original MP1 stain showing only DAPI and CD8 staining, followed by the same image with CD8+ detection applied in v0.2.0-m4 and v0.2.0-m9 method one. CD8+ cells are seen in magenta and negative cells in grey. Bar graph displays the percentage of CD3+/CD8+ cells detected in the lymphoid cores (n = 10) using v0.2.0-m4 and v0.2.0-m9 method one. The only significant difference in cell positivity is in lymph node tissue (p=0.0162). (C) From left to right: Composite image of the original MP1 stain, cell detection with v0.2.0-m4, cell detection with v0.2.0-m9 method one showing exclusively the ‘m4’ classes (3^rd^ image) and then the additional classes (4^th^ image). These classes are all listed in (D), followed by the same stain with overlaying cell detection from v0.2.0-m9 method two, again portraying only the ‘m4’ classes (3^rd^ image) and then the additional classes (4^th^ image).
